# Biological fluid dynamics of airborne COVID-19 infection

**DOI:** 10.1007/s12210-020-00938-2

**Published:** 2020-08-16

**Authors:** Giovanni Seminara, Bruno Carli, Guido Forni, Sandro Fuzzi, Andrea Mazzino, Andrea Rinaldo

**Affiliations:** 1 Accademia Nazionale dei Lincei, Rome, Italy; 2 Università di Genova, Genoa, Italy; 3 Istituto di Fisica Applicata Nello Carrara (IFAC), Consiglio Nazionale Delle Ricerche, Sesto Fiorentino, Italy; 4 Istituto di Scienze dell’Atmosfera e del Clima (ISAC), Consiglio Nazionale Delle Ricerche, Rome, Italy; 5 Dipartimento di Ingegneria Civile, Chimica e Ambientale (DICCA), Università di Genova, Genoa, Italy; 6 Istituto Nazionale di Fisica Nucleare, Via Dodecaneso 33, 16146 Genoa, Italy; 7 Laboratory of Ecohydrology IEE/ENAC, École Polytechnique Fédérale de Lausanne, Lausanne, Switzerland; 8 DICEA, Università di Padova, Padua, Italy

**Keywords:** Respiratory emissions, Coughing, sneezing, speaking, Clouds, jets, puffs, Droplets, Distancing, Non-pharmaceutical protection measures

## Abstract

**Abstract:**

We review the state of knowledge on the bio-fluid dynamic mechanisms involved in the transmission of the infection from SARS-CoV-2. The relevance of the subject stems from the key role of airborne virus transmission by viral particles released by an infected person via coughing, sneezing, speaking or simply breathing. Speech droplets generated by asymptomatic disease carriers are also considered for their viral load and potential for infection. Proper understanding of the mechanics of the complex processes whereby the two-phase flow emitted by an infected individual disperses into the environment would allow us to infer from first principles the practical rules to be imposed on social distancing and on the use of facial and eye protection, which to date have been adopted on a rather empirical basis. These measures need compelling scientific validation. A deeper understanding of the relevant biological fluid dynamics would also allow us to evaluate the contrasting effects of natural or forced ventilation of environments on the transmission of contagion: the risk decreases as the viral load is diluted by mixing effects but contagion is potentially allowed to reach larger distances from the infected source. To that end, our survey supports the view that a formal assessment of a number of open problems is needed. They are outlined in the discussion.

**Graphic abstract:**

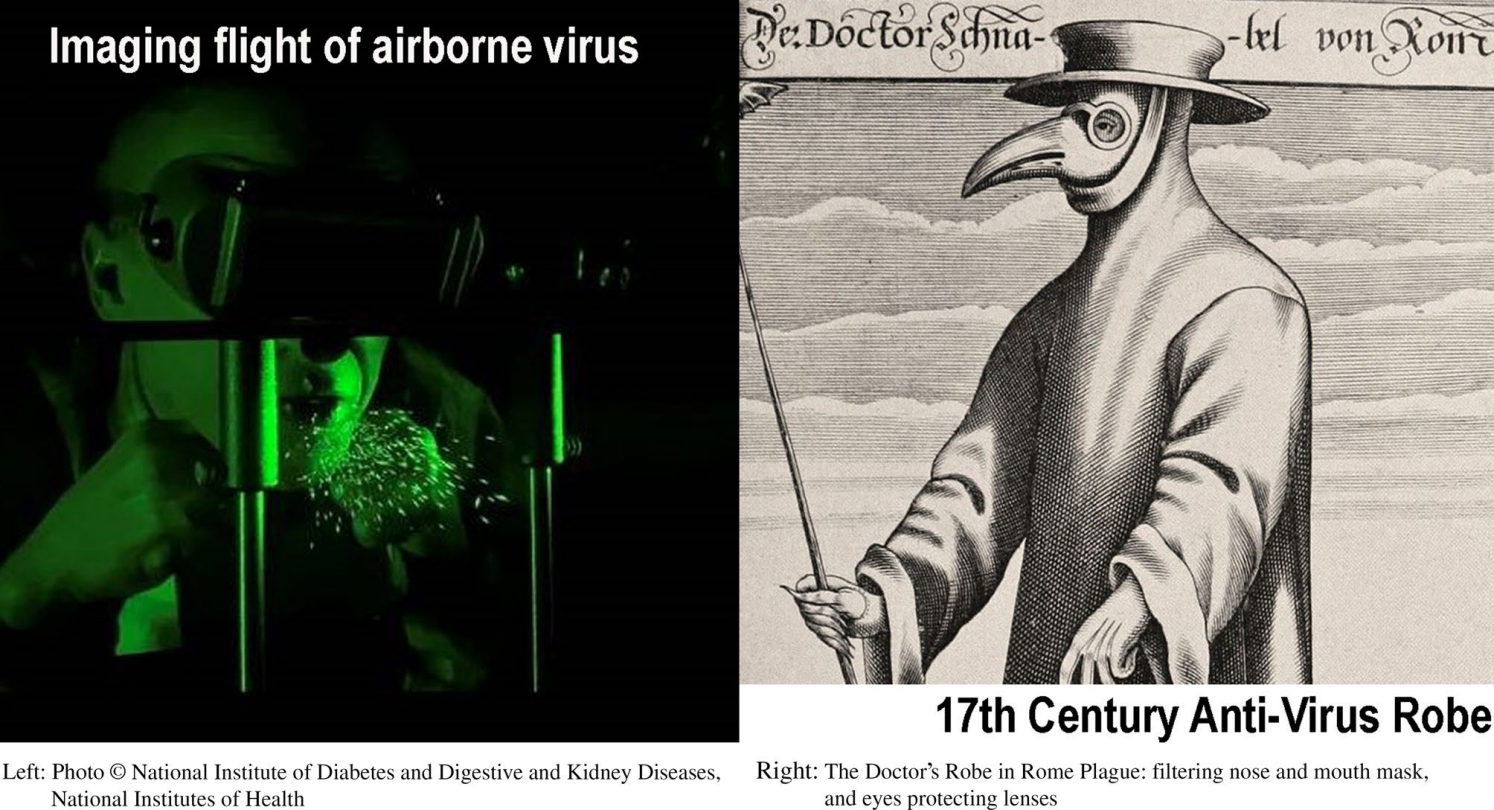

Whether generated by rotting matter or emanating from infected persons, animals, or objects, the venomous atoms would infect salubrious air and make it “miasmatic”—that is, poisonous. It was indeed the “corruption” of the air that, according to the doctors of the Renaissance, was the basic precondition for the outbreak of an epidemic of plague.

Fighting the Plague in Seventeenth Century Italy, The University of Wisconsin Press, Madison 1981, p. 8. (Carlo Cipolla, 1922–2000, Linceo from 1987). (photo credit: National Institute of Diabetes and Digestive and Kidney Diseases, National Institutes of Health.)

## Introduction

In a report published by the US National Academy of Sciences (NAS) on the reuse of facemasks for the protection against respiratory viral infection, it is stated that:

The public is likely to forgive lack of knowledge but will not be willing to trust public health officials in the next instance if they have in any way been misinformed or misled (National Academy of Sciences of the United States, 2006, p. 67)

This statement poses an important question, that of information and citizens’ right to knowledge. Bearing this in mind, the Accademia Nazionale dei Lincei Committee on the Environment and Great Natural Catastrophes has decided to produce a report on an aspect of the current pandemic that deserves attention, not only by the Institutions responsible for managing the pandemic but also by research institutes.

**Fig. 1:**
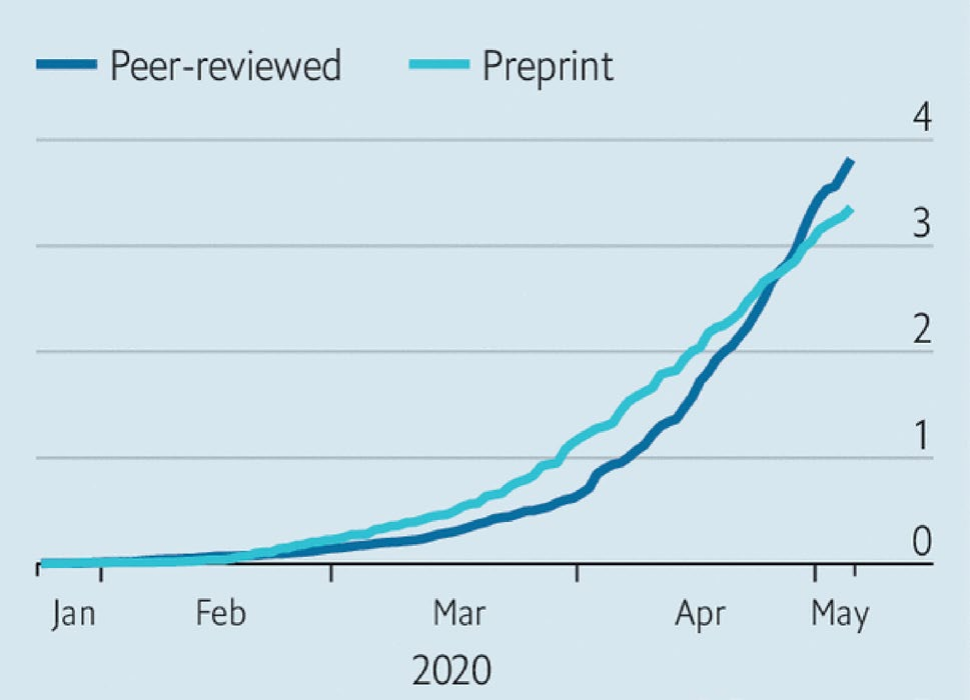
Upsurge in research on various aspects of the pandemic: cumulative number of research studies (in thousands) published from January to May 2020 (source: The Economist, May 7, 2020). https://www.economist.com/science-and-technology/2020/05/07/scientific-research-on-the-coronavirus-is-being-released-in-a-torrent

The question we propose to examine is the state of knowledge on the mechanisms involved in the airborne transmission of SARS-CoV-2 infection, with particular reference to the important contribution that can be gained to the development of this knowledge from the interaction between immunologists, virologists and biofluid dynamic experts. However, some preliminary clarifications are needed. The present review does not (nor it reasonably could) claim of being exhaustive. Indeed, the question in hand certainly is not new (if not for the particular pathogen involved): many researchers, not only in the biomedical fields, have been reporting on this topic for decades. Moreover, the explosion of related research publications on SARS-CoV-2 is of unimaginable numbers, as shown in Fig. 1.

We just aim to demonstrate how even a partial examination of the relevant literature highlights important unresolved issues, suggesting research paths to be pursued. An important clarification concerns practical recommendations regarding personal protection measures and equipment to prevent the contagion. They are discussed in Sect. 5 and in our conclusions. Nonetheless, this is done with the awareness that science is responsible for providing the framework of knowledge, and policy to assess the risks and possible decisions while respecting the necessary distinction of roles and transparency regarding the participation of science and politics.

The open problems where fundamental research, either theoretical or experimental, is needed are conveniently grouped into three distinct classes.

The first-class concerns the precise characterization of the ‘cloud’ emitted through the varieties of respiratory emissions as a likely mode of disease transmission. Observations confirm that a sizable probability exists that normal speaking causes airborne virus transmission, especially in confined environments. The modeling of droplet generation mechanisms, that underpin potential contagion through destabilization of the mucus layer that covers the respiratory tract, is still hardly mature for generalized applications. Somewhat surprisingly, it emerges that currently experimental findings do not allow us to single out the proper probability distribution of the size of the droplets, that can be associated with the various respiratory emissions. Despite a research effort that has spanned over a century and the use of progressively more refined experimental techniques, still the various studies provide results that can differ broadly, even by orders of magnitude. Recent developments based on ultra-rapid image processing techniques suggest that, at least in the case of violent emissions (coughing, sneezing), the process of droplet formation and dispersion continues in the first phase of expulsion through the fragmentation of liquid sheets and filamentous structures, whose modeling poses a complex problem of fluid dynamics.

The second class of problems concerns our understanding of the transport processes through which the cloud modifies its composition on moving away from the source. These modifications affect the possible infection mechanisms. Larger droplets tend to settle in the immediate vicinity of the infected emitter, while others are advected away from the source and evaporate at rates dependent on temperature and relative humidity of the emitted clouds. Initially warmer and more humid than the external environment, clouds undergo mixing with the turbulent ambient air. Therefore, the evolution of velocity, temperature and relative humidity fields and their turbulent fluctuations ultimately determines the particle size distribution of the droplets. In the distant field, evaporation reduces the surviving droplets to their dry nuclei.

The third class of open problems originates from a simple question: does the infected droplet that undergoes evaporation, possibly shrinking to its dry core, remain infectious? This is tantamount to addressing the general problem of the prediction of the stability of viruses in environments characterized by different temperature and humidity conditions. Even in this domain, science does not seem to have reached an assessment to date. Our report draws together several lines of argument about the persistence of SARS-CoV-2 infectivity in the environment. Given the relevance of this problem, however, it seems surprising that even the fundamental mechanisms that determine the rates at which viruses do no longer retain their infectivity in the air (e.g. the role of possible coating, dissolved salts, and pH variations, to name a few) have not been conclusively assessed to date.

We also focus on the fluid dynamic background underlying measures and equipment for personal protection from the contagion. Remarkably, the analysis of recent visualizations of the respiratory emissions by individuals wearing protective masks highlightsthe limits and validity of the use of personal protection equipment like face masks and eye protections. An important finding that emerges clearly is that the one-meter measure of social distancing recommended by the World Health Organization (WHO) is not based on direct scientific evidence, nor is truly conservative. This notwithstanding, the effectiveness of social distancing for risk reduction is undisputable, especially if accompanied by suitable use of protective equipment: in their absence, the benchmark safe distancing of 1 m, the WHO standard, appears largely insufficient.

This review finally examines the bio-fluid dynamic underpinning of epidemiological models, strongly supporting the scientific, social and economic importance of strengthening interdisciplinary research on this topic. It is organized as follows: Sect. 2 outlines various issues related to the biology of the contagion, with subsections focusing on: a description of the virus (Sect. 2.1); the physical-biological barrier protecting the cells of the mucosa of the respiratory tract from the spread of the infection (Sect. 2.2); mechanisms of virus penetration into the human cell (Sect. 2.3); generalities on the disease (Sect. 2.4). Section 3 deals with an overview of the exposure mechanisms, specifically centered on: direct contact and airborne transmission (Sect. 3.1); the rationale behind, and the limits of, commonly employed distinctions between large and small droplets carrying viral loads (Sect. 3.2); related issues on the uncertainty of the roles of ‘close’ or ‘distant’ infection mechanisms (Sect. 3.3). Section 4 narrows down to specific fluid dynamics problems arising in airborne transmission, subdivided into subsections on features of particles or filaments emitted by the respiratory activities (Sect. 4.1); experimental evidence (Sect. 4.2); droplet formation mechanisms (Sect. 4.3); open problems in the fluid dynamics of the two-phase flow of expiratory events (Sect. 4.4); the roles of natural and forced ventilation (Sect. 4.5). Section 5 focuses on the fluid dynamics of the protection from airborne infection transmissions. Its subsections deal with measures for facial protection (Sect. 5.1); and the fluid dynamics of so-called social distancing (Sect. 5.2). Section 6 concentrates on the fluid dynamics implications for the development of epidemiological models.

A set of conclusions, subsumed by six open questions whose relevance is discussed, closes the paper (Sect. 7).

## Biology of the contagion

Although this review focuses on the fluid dynamics of the contagion, some information on its pathology must also be provided.

### The virus

**Fig. 2:**
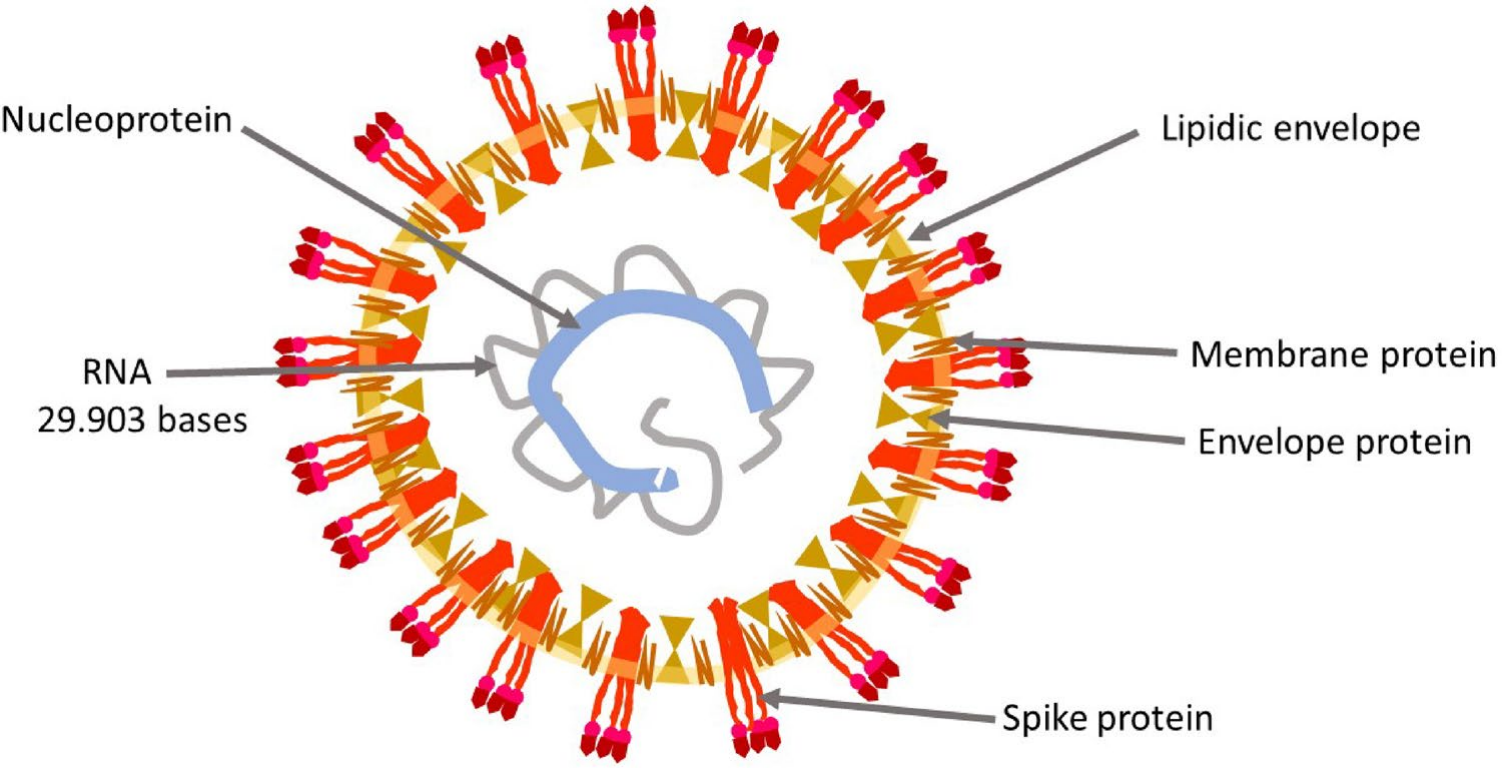
Schematic illustration of the structure of SARS-CoV-2 virion

The second CoronaVirus causing a Severe Acute Respiratory Sindrome (SARS-CoV-2, Fig. 2) is an oily spherical particle, with a 0.125 (0.05–0.2) μm (micron) diameter. The outer shell (the pericapsid) consists of three structural glycoproteins: Spike, Envelope, and Membrane and a lipid coating. The large Spike protein (S), which protrudes on the outside layer, consists of two domains, S1 and S2. The most external S1 domain, a region known as RBD (Receptor Binding Domain), contains an area which allows the binding of the virus to human cells. On the surface of SARS-CoV-2, three S glycoproteins aggregate to form a homotrimer. Numerous homotrimers protruding outside the pericapsid give rise to a crown-like appearance, hence the name Coronavirus (Walls et al. 2020). Inside the pericapsid there is a single-stranded positive-sense RNA of about 30 kb containing 30 000 bases, a very large RNA virus genome (Chan et al. 2020). A forth virus structural protein, the Nucleocapsid protein, wraps and coils virus RNA, keeping it stable inside the pericapsid.

### Physical-biological barrier

Although little is known on the infectious load of SARS-CoV-2, which exerts an important influence on the contagion, it is supposedly low, between 10 and 1000 viral particles transmitted via the respiratory system (Cyranoski 2020).

**Fig. 3:**
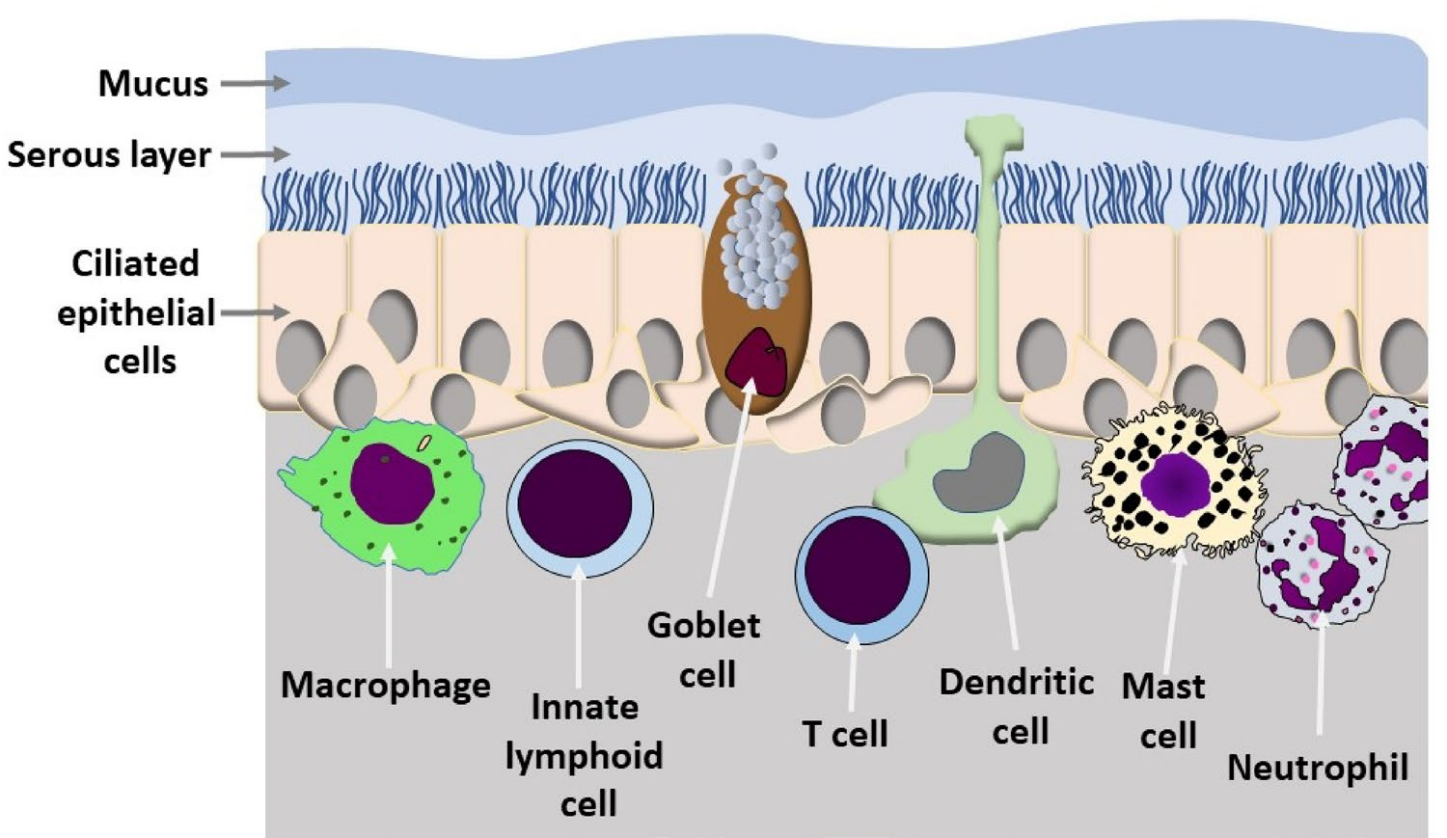
Schematic of the mucociliary barrier providing the first defense against respiratory pathogens

The mucus, a viscous gel that covers the cells of the mucosa of the respiratory tract, harnesses and neutralizes the viral particles and thus prevents contact with the surface of the host cells. The mucus is a complex mixture of glycoprotein continuously produced by goblet cells of the mucous membranes and from particular glands. The mucus also contains salts, lactoferrin, enzymes and antibodies (secretory IgA and IgM) (Birchenough et al. 2015). The production of mucus is mainly regulated by two lymphokines (IL-13 and IL-22) secreted by sentinel lymphocytes associated with the mucosal barrier (Fig. 3). IL-13 is produced primarily by Innate Lymphoid Cells (ILC), IL-22 by T-helper 17 cells. The overproduction of mucus gives rise to phlegm (Toki et al. 2020). The mucus is continuously transported by the cilia of epithelial cells of the mucosal membranes (Fig. 3), it is swallowed and destroyed in the stomach. The movement of the mucus is fundamental for its protective action (see also Sect. 4.3). In normal conditions, the ciliary beat frequency is around 700 beats per minute. The intensity of the beats is regulated negatively by the IL-13 and is lowered by environmental pollutants present in the air we breathe and by the air humidity and low temperature (Laoukili 2001). The low relative humidity and the low temperature of the air we breathe alter both the production and the composition of the mucus. Low air temperature also reduces the functionality of the immune system cells associated with respiratory mucociliary membranes (Moriyama et al. 2020). How the environmental conditions influence the protective barrier against SARS-CoV-2 has not yet been reported, although the influence that humidity, temperature and pollution in general have on the seasonality of coronavirus infections, which cause the common cold, is well known.

### Penetration into the human cell

**Fig. 4:**
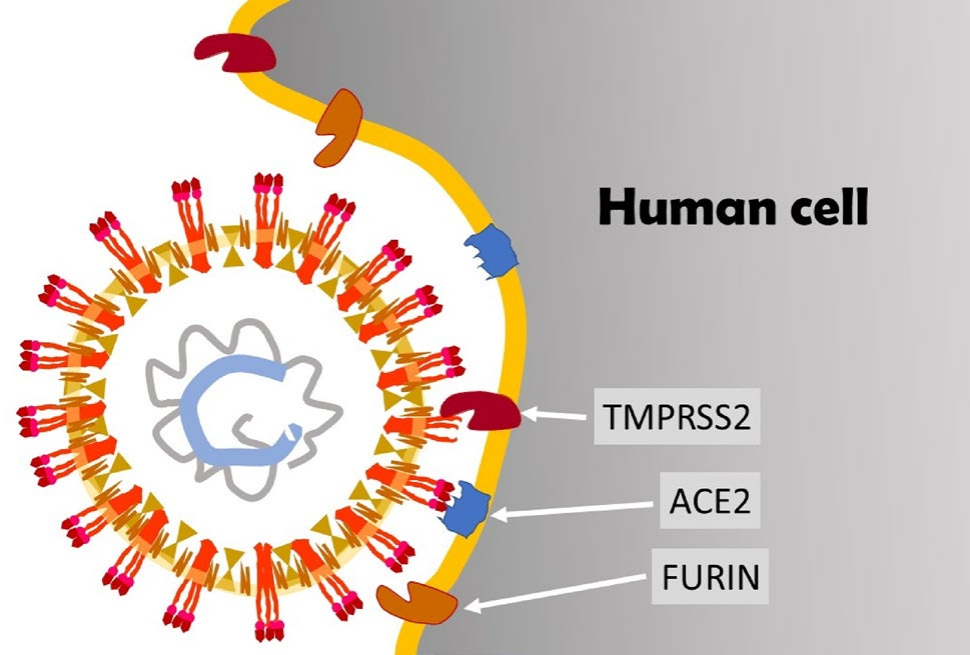
Schematic representation of the interaction between SARS-CoV-2 and the human cell membrane

If the viral particles overcome the mucus barrier and reach the surface of the cells of the respiratory mucosa the RDB zone, present in S1 domain of Spike protein, will bind with high affinity to the N-terminal domain of ACE2 (*Angiotensin-Converting Enzyme 2*), an exopeptidase that catalyzes the conversion of angiotensin from vasoconstrictor to vasodilator. This enzyme is normally present on the membrane of many human cells (Shang et al. 2020) (Fig. 4).

Once the virus is docked to the cell, other proteolytic enzymes present on the cell membrane, such as TMPRSS2 (*TransMembrane PRotease Serine 2*) and Furin, remove the outer part of Spike proteins, separating the S1 domain from the S2 domain. Following this separation, the S2 domain on the external surface of the virus exposes particular sequences of amino acids (*fusion peptides*) that facilitate the fusion between the viral pericapsid and the membrane of the human cell (Cyranoski 2020; Shang et al. 2020). Thanks to this fusion, the RNA of the virus can penetrate into the cell and is immediately translated into proteins by host cell ribosomes. The infected cells then die releasing millions of new viral particles that begin to invade other cells and cause the COVID-19 disease.

### The disease

The clinical characteristics of the COVID-19 disease stem from the competition between the invasive action of the virus and the immune reaction. The viral load, its decimation by the reaction of the mucociliary barrier, the immediate innate immune response and delayed adaptive immune response determine whether the disease will be asymptomatic, mild or severe (Matricardi et al. 2020). There are many aspects to this competition, often the reaction leads to ambivalent results, in the sense that a reaction aimed at inhibiting viral expansion can lead to events which will promote or worsen the course of the disease (Matricardi et al. 2020). During the course of the infection the virus will spread from the nasal and tracheal epithelium to the lungs. With greater or lesser effectiveness immune reactions hinder or block the virus spreading. The direct transmission of the SARS-CoV-2 to the pulmonary alveoli could also be favored by deep breathing, as it would occur with intense athletic exercise (Matricardi et al. 2020).

Once it reaches the lung, the SARS-CoV-2 infects and kills the alveoli cells that abundantly express ACE2 and carry lots of ACE2 receptor enzyme on their surface. In the lungs, the violent immune reaction that is triggered against viral infection contributes significantly to the onset of a severe respiratory insufficiency, known as ARDS (Acute Respiratory Distress Syndrome). ARDS, together with disseminated intravascular coagulation, represent the most severe complications of the COVID-19 disease.

## Possible mechanisms of exposure to infection

It is commonly agreed that SARS-CoV-2 is highly infectious. The reason for this is still not entirely clear. It is an important question, that cannot be resolved solely by epidemiological studies since it requires first a clear understanding of the possible mechanisms of exposure to the virus.

### Direct contact and airborne transmission

The airborne transmission, the dominant route of SARS-CoV-2 spread, does not necessarily involve a physical contact between the infected and the susceptible persons. The virus is transmitted mainly via small respiratory droplets containing the viral particles that the infected person exhales when coughing, sneezing or talking. The amount of virus released increases as the infection progresses. The amount of virus spread by an infected person who is asymptomatic is significantly lower than that of a COVID-19 patient with symptoms (Ferretti et al. 2020). However, the magnitude of the transmission of SARS-CoV-2 by infected asymptomatic people is difficult to assess, although numerous data from China (Day 2020), as well as studies conducted in Vo' Euganeo, Italy (Lavezzo et al. 2020) and in Iceland (van Doremalen et al. 2020) reveal that a large percentage of the infected population is asymptomatic. It is, therefore, likely that asymptomatic and presymptomatic individuals are the largest source of infection.

The virus spread by an infected person can also be deposited on various surfaces. It is, therefore, possible to transmit the infection also through contact between a susceptible and an infected person, either directly (e.g. hand shaking, one of which is infected) or indirectly, through fomites (e.g. a contaminated handkerchief). Infection takes place if the susceptible individual touches his/her mouth, nose or eyes after picking up the virus by touching an infected surface (Wang et al. 2020).

Two aspects play a fundamental role in assessing the importance of the different mechanisms of infection: knowledge of the viral load that could potentially be released via respiratory droplets, and knowledge of the persistence of the viral infectivity over time, in relation to the variations in environmental conditions. Unfortunately, the present state of knowledge on these two aspects does not allow us to make conclusive statements.

Viral load analysis has been performed by Wölfel et al. (2020) on nasal, oropharyngeal and sputum swabs of SARS-CoV-2 patients. The viral load recorded was dependent on the time that had elapsed since the onset of the symptoms. In salivary secretions, the virus load was around 7 × 10^6^ virions per milliliter, with peaks exceeding 2 × 10^9^ virions per milliliter.

Results on the persistence of viral infectivity of SARS-CoV-2 virus have been reported by van Doremalen et al. (2020) and Chin et al. (2020). The first group assessed the stability of virus infectivity in aerosols maintained at 21–23 °C and relative humidity of 40%. The results show a persistence of virus infectivity for the entire duration of the experiment (3 h), albeit with a decrease in viral load by a factor of about 6, a decrease that is similar to that found for SARS-CoV. Chin et al. (2020) measured the stability in the laboratory of SARS-CoV-2 maintained at different temperatures. Tests for its infectivity were performed after a 14-day incubation period. The virus was very stable at 4 °C, showing a reduction of the infectious load by a factor of 5 on day 14. Increasing the temperature to 70 °C, the virus became inactive within 5 min.

Previous studies have been conducted on both the MERS-CoV, the coronavirus which caused the Middle East Respiratory Syndrome—MERS—in 2012 (Pyankov et al. 2017) and HCoV 229E coronavirus (Geller et al. 2012). In the first case, 63% of the virus nebulized in particles between 1 and 2 μm remained infectious after one hour at 25 °C and 79% relative humidity, whereas particles kept for one hour in the warmer and drier environment (38 °C and 24% relative humidity) reduced the virus infectivity to less than 5%. The second study reported data on the persistence of viral infectivity of the aerosolized HCoV229E virus, kept for a period ranging from 15 min to 6 days at 20 °C in the presence of different relative humidity levels. After 15 min with 80% relative humidity, only 55% of the viral particles remained infective. On the contrary, with 30% and 50% relative humidity, 90% and 87% of the viral particles remained infective. After six days, 20% of the viral particles kept at 30% relative humidity were still infectious, while there was no infectious activity in the particles kept at 80% or 50% relative humidity. The influence that the relative humidity and temperature have on the virus is evident in the study by Ijaz et al. (1985), which shows that in a 20 °C environment the infectivity of the HCoV 229E coronavirus remains very high in the presence of 50% relative humidity, while it decreases significantly with both 30% and 80% relative humidity. When the temperature is lowered to 6 °C, the virus remains highly infectious at both 50% and 30% relative humidity, while it loses its infectivity at 80% relative humidity.

These results reveal a partly contradictory and inconsistent response: MERS-CoV seems to not endure a hot and dry climate, while SARS-CoV-2 and HCoV 229E viruses remain stable in low humidity environments. However, these data suggest that changes in relative humidity drastically affect the infectious power of the coronaviruses. This effect is evident both at temperatures around 20–25 °C and at lower temperatures. Further data are necessary to establish the survival of SARS-CoV-2 in different environmental conditions and to predict the seasonality of the infection (see the recent review by Moriyama et al. 2020 on the seasonality of coronaviruses). However, the general observation remains that other viruses, such as polio, Sabin strain, appear much more fragile than the coronaviruses.

As far as the stability of the viruses on surfaces, van Doremalen et al. (2020) analyzed the persistence of infectivity of SARS-CoV-2 on different surfaces and observed that their stability is higher on plastic and stainless-steel surfaces than on copper and cardboard. On plastic (steel) surfaces the virus retained its infectivity for 72 (48) h, with viral load reduction by a factor greater than a thousand. SARS-CoV showed similar results. On copper (cardboard) surfaces the viral load had disappeared after 4 h (24) h. In a similar study by Chin et al. (2020) a 5 μL droplet of virus culture was pipetted on a surface and left at room temperature (22 °C) with relative humidity around 65%. No infectious virus was detected after 3 h, and 2, 4, and 7 days, respectively on paper, wood-fabrics, glass and banknotes, stainless steel and plastic surfaces. In addition, a measurable level of infectious virus (around 0.1% of the initial value) was still present on the outer surface of a surgical mask after 7 days.

**Fig. 5:**
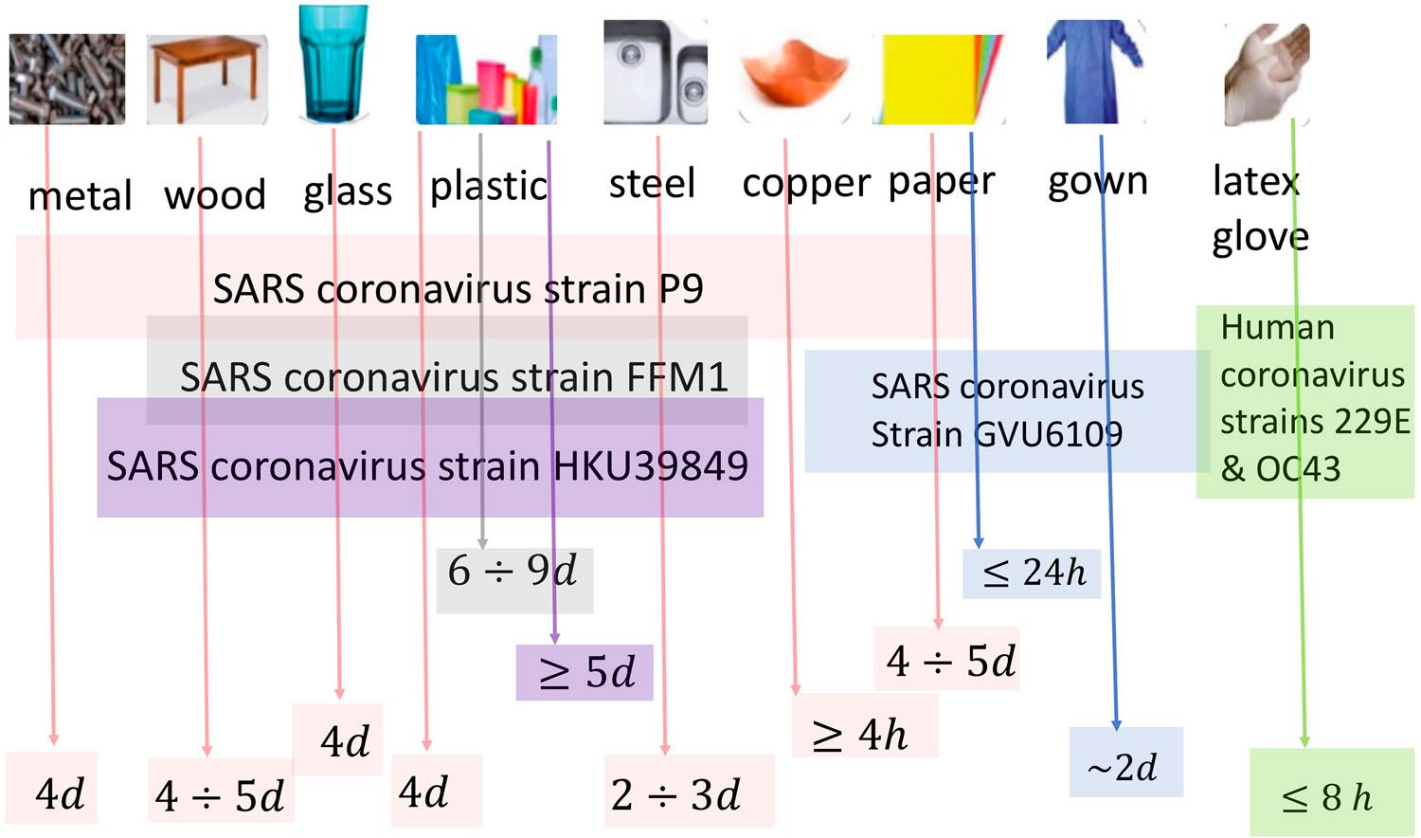
The sketch illustrates the results from the scientific literature on the persistence of different viruses on various surfaces (adapted from Fathizadeh et al. 2020)

An outline of the findings on the stability of coronaviruses on various types of surfaces is reported in Kampf et al. (2020). Results are illustrated schematically in Fig. 5 (Fathizadeh et al. 2020). A comparison with results obtained from the SARS-CoV-2 suggests that survival of the virus strongly depends on the specific characteristics of the virus, as well as on the environmental conditions.

### Large droplets and small droplets

In the epidemiological literature (e.g. Asadi et al. 2020, Service 2020) it is suggested that in airborne transmission there are two mechanisms involved:

- ‘Close’ infection associated with large droplets, in close proximity to the infected person
- ‘Distant’ infection associated with small droplets, which can remain airborne for a very long period of time and, therefore, can reach a 'large' distance from the infected person (how large will be discussed later in this review). When inhaled, particles of this size can directly reach the deep part of the respiratory system. Indeed, their presence has been clearly ascertained with the SARS-CoV-2 in the bronchoalveolar lavage fluid of infected people in Wuhan and Beijing (Zhu et al. 2020).

It may be surprising to learn that the dichotomous classification of airborne transmission: ‘big’ droplets—‘small’ droplets, with the implication of 'close' versus 'distant' infection, dates back to an important and much cited study on tuberculosis transmission published almost a century ago (Wells 1934). The classification was based on the following criterion:

- The 'large' droplets settle quickly and thereby do not undergo total evaporation. Therefore, the infection can take place only in the area close to the infected person;
- The 'small' droplets, pass from the hot and humid environment of the respiratory system of the infected person to the colder and less humid external environment and thereby evaporate rapidly and are transformed either into dry residual particles, the ‘nuclei of the droplets’, or into smaller liquid droplets in equilibrium with the ambient. This latter possibility is allowed owing to the solute effect allowing equilibrium between salty liquid water (a model for saliva) and its vapor even under unsaturated ambient conditions.

Thus, two important players are involved in the process: droplet sedimentation and droplet evaporation. The time scale of sedimentation can be easily estimated by referring to the simplest case, that of a 'small' rigid sphere that deposits in an otherwise stationary fluid. This approximation obviously ignores the possible reduction in diameter induced by droplet evaporation. In the low Reynolds number approximation, the settling speed *w*_s_ of a rigid sphere is given by Stokes formula *w*_s_ = *g* *s d*^*2*^/18 $\nu$_a_, with *d* particle diameter, $\nu$_a_ kinematic viscosity of the air (= 1.5 10^−5^ m^2^/s at 20 °C)*, g* gravity and *s* relative density of the droplet in the air (*s* = 816 at room temperature). Hence, droplets with a diameter equal to 100 μm settle with a speed of about 0.3 m/s, corresponding to a Reynolds number equal to 2, for which the Stokes approximation would not be strictly valid but is still sufficient for our illustrative purposes. Note that the settling time increases very rapidly as the particle diameter decreases, hence the smaller is the droplet the higher is the role of evaporation. By contrast, the evaluation of the time scale of the evaporation process is not so immediate. Evaporation results from heat and mass exchange at the droplet–air interface. Within a purely diffusive scheme (stationary gas phase), both these processes originate from the existence of a gradient (temperature and vapor concentration respectively) at the interface. Indeed, the droplet coming from the respiratory system is warmer than air, the vapor concentration in the gas at the interface is higher than the vapor concentration at a distance from the droplet. Motion of the gas phase gives rise to convective effects, associated with both the mean flow field and the turbulent fluctuations if the air motion is turbulent, for which an estimate of the droplet-air relative velocity is difficult. The latter important aspect was neglected by Xie et al. (2007) (see discussion in Sect. 4.4).

**Fig. 6:**
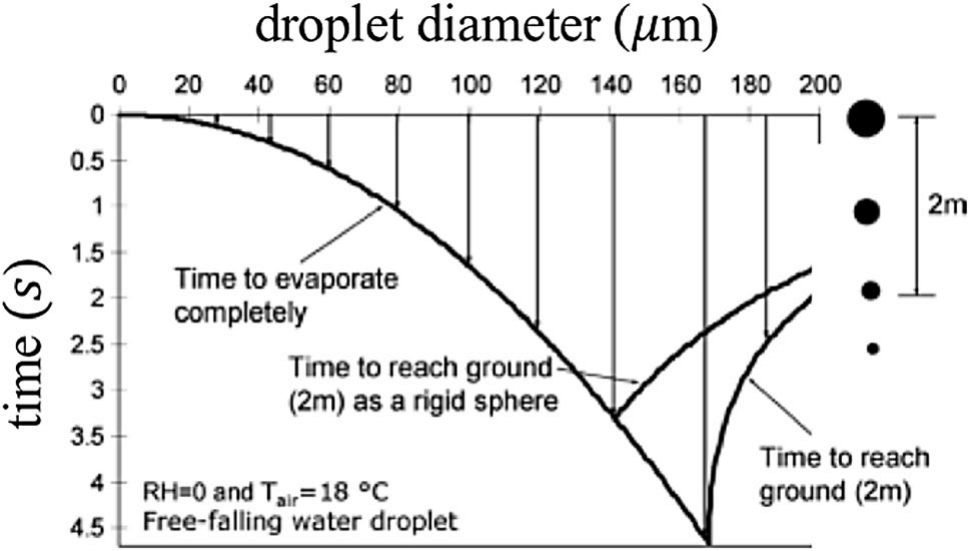
A plot of the sedimentation-evaporation of the droplets (Wells 1934), providing an estimate of the time scale of the two processes as a function of the droplet size (adapted from Xie et al. 2007)

Wells (1934) was the first to attempt an estimate of the evaporation time in the epidemiological context, although the procedure he used to obtain the result plotted in Fig. 6 is not wholly clear (Xie et al. 2007). From his model, Wells (1934) concluded that, under ordinary conditions, droplets smaller than 100 μm evaporate completely before depositing. As a result, ‘close' infection would be associated with droplets larger than 100 μm. His study also showed the mechanism by which droplets are transformed into dry 'nuclei'.

Surprisingly, for over 70 years, the foundation of this study has been accepted by the epidemiological community without any attempt to re-examine the analysis, despite the fact that the problem of droplet sedimentation-evaporation has received great attention in many other fields, from cloud physics, to combustion, spray technologies, ink-jet printing, just to name a few. Only recently, Wang et al. (2005), Xie et al. (2007) and, subsequently, Ghaem-Maghami and Johari (2010) have revised Wells' findings using physical models able to account, even if in a simplified manner, for most of the phenomena that play some role. In particular, they have introduced a third ingredient in the formulation, namely the convective effect of the fluid which transports the particles. The latter was treated as a jet, an assumption that is inappropriate, as discussed in Sect. 4.4.

**Fig. 7:**
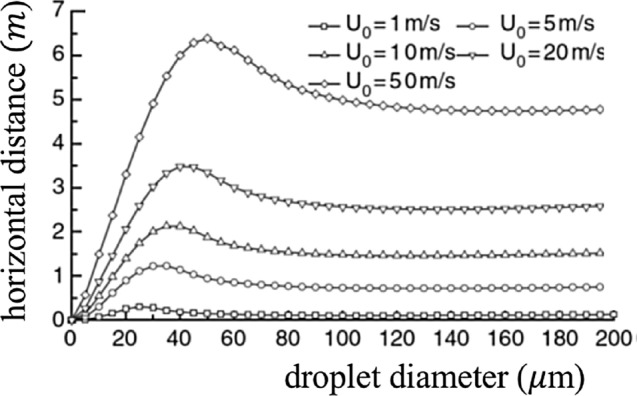
Horizontal distances reached by droplets of various sizes as the initial speed *U*_0_ of the expiratory jet increases according to Xie et al. (2007) (adapted from Xie et al. 2007)
- Finally, the simulations show that, as the relative humidity decreases, the size of the particles that reach a distance of 2 m before evaporation decreases. In effect, as the relative humidity decreases, the evaporation process is enhanced and the number of particles that evaporate completely increases. This would suggest that low ambient humidity favors the airborne spread of the infection, confirming the results by Wang et al. (2005). Indeed, as discussed in Sect. 3.1, coronavirus infectivity is drastically affected by the mutual interaction between relative humidity and temperature.

The picture arising from the study by Xie et al. (2007) confirms, but also corrects the outcome of Wells (1934) analysis. In particular:

- If convective effects are neglected, then results confirm the existence of a critical droplet size *d*_c_ below which the droplet evaporates completely before settling. The critical value of the droplet size depends on the relative humidity of the air (*H*_*r*_*)* and is greatly reduced as *H*_*r*_ increases (*d*_c_ = 125 μm, 100 μm, 85 μm and 60 μm, for *H*_r_ = 0%, 50%, 70% and 90%, respectively). These values are however lower than those predicted by Wells (1934).
- Taking into account the convective effect associated with the mean flow, the picture is further modified. The larger droplets leave the stream quickly and settle; the intermediate droplets leave the stream and evaporate totally before settling; the smaller droplets are transported by the stream until they evaporate completely and become dry nuclei. Moreover, the horizontal distance travelled by the droplet before settling (or evaporating) is strongly dependent on the initial speed of the cloud (hence on the type of expiratory emission), as well as on droplet size and relative humidity.

- Fig. 7 shows that the diameter of the droplets that reach the maximum distance increases from 30 μm to 50 μm as the initial speed increases from 1 to 50 m*/s*, respectively. Furthermore, the droplets do not reach distances exceeding 1 m with a stream velocity of 1 m*/s* (normal breathing), but do reach distances of more than 6 m with a stream velocity of 50 m/s (sneezing)!

Note that these conclusions are based on the assumption that a dry residual nucleus of a droplet retains its infective capacity. As will be illustrated in Sect. 4.4, they are also crucially dependent on the neglected role that the turbulent character of two-phase expiratory flows can play on the evolution of the humidity field and, therefore, of the droplet spectrum.

### ‘Close’ or ‘distant’ infection transmission?

The scientific community has not reached a unanimous consensus on the relative importance of the various mechanisms of infection transmission for several infective diseases. In particular, the role of airborne transmission has been emphasized for the flu, tuberculosis, cold and whooping cough (Fennelly et al. 2004; Tellier 2006, 2009; Atkinson and Wein (2008; Fabian et al. 2008; Clark and de Calcina-Goff 2009), Tellier et al. 2019). Studies performed on SARS indicate the importance of both ‘close’ and ‘distant’ transmission (e.g. Wong et al. 2004; Yu et al. 2004). As for SARS-COV-2 the state of knowledge is quite uncertain (Lewis 2020). In a few recent preprints (Liu et al. 2020; Santarpia et al. 2020) the presence of viral RNA from SARS-CoV-2 has been reported in aerosols collected in various locations of Wuhan’s hospital and its neighborhoods, as well as in air and surface samples in a hospital in Nebraska hosting COVID-19 patients. Indirect mechanisms thus support the use of airborne isolation precautions when caring for COVID-19 patients. It must be underlined, however, that the presence of viral RNA does not imply the presence of the infectious virus.

The uncertainty on the mechanism of infection transmission is not surprising. Indeed, resolving the ‘close’—‘distant’ dilemma requires that a bunch of important issues, that complicate the picture outlined in the previous sections, be preliminarily tackled. They concern the fluid dynamics of the variety of processes whereby the virus moves from the airways of the infected individual to its susceptible target. How do expiratory emissions form within the respiratory system? And how do they transform into the system of droplets contained in the two-phase flow? What is the grain size distribution of droplets? Can one identify a threshold value of the grain size, to allow a scientifically sound distinction between large and small droplets? How does the two-phase flow evolve and how is its structure affected by turbulence? We will review the state of the art on these issues in Sect. 4 considering both violent expiration events (cough and sneeze) and normal ones (breath and speech). Indeed, it has been known for some time and has been confirmed by recent studies (cfr. Sect. 4.1), that in the normal breathing and speaking functions, a large number of aerosols is released in the air. They consist of small droplets, invisible to the naked eye, and easily inhaled, which are however large enough to host virus particles. This feature may help explaining whether this mode of virus transmission is responsible for the role apparently played in the SARS-CoV-2 transmission by asymptomatic or weakly symptomatic individuals who, by definition, do neither cough nor sneeze. In this respect, note that the epidemiological model of Li et al. (2020) suggests that roughly 86% of infected individuals in Wuhan, prior to lockdown implementation, had not been tested because they were either asymptomatic or weakly symptomatic.

A last crucial issue is that of ascertaining to what extent these processes are affected by the environmental conditions, i.e. air temperature and humidity, as well as by the presence of ambient air motion driven by natural or forced ventilation. A few hints on these aspects are given in Sect. 4.5.

## Fluid dynamics problems arising in airborne infection transmission

Below, we follow an inductive approach by letting open problems emerge from a review of the progressive development of research. Transmission of COVID-19 could be summarized as follows. Human atomization of viruses arises from coughing or sneezing of an infected person, producing a spectrum of virus-containing droplets. Virus transmission from person to person occurs via the variety of direct/indirect contacts and airborne aerosol/droplet routes (say, via nascent aerosols from human atomization). Large droplets settle relatively close to the source to cause person/object contamination when distancing is limited, while aerosols that disperse in the air may cover much larger distances. Needless to say, a clear-cut dichotomy among the transport properties of ‘large’ vs. ‘small’ particles is overly simplistic. Direct and airborne transmissions thus occur at the variable range and extended time, respectively. Inhaled airborne viruses deposit directly into the human respiration tract (Zhang et al. 2020). Overall, the prevailing view (although not yet universally accepted) is that airborne transmission is highly virulent and represents the dominant route of spread of the disease.

### Key features of the particles emitted by the respiratory activities

The seminal papers of Flügge (1897) and Wells (1934) have highlighted since the ‘40 s of the past century, the importance of better characterizing the size distribution of the droplets emitted by expiratory events. It is somewhat striking to note that the early study of Duguid (1946) had motivations quite similar to those that inspire the current investigations, including the possible role of asymptomatic individuals: “…*The expiratory activities which have been considered productive of droplet-spray, are sneezing, coughing, speaking, laughing and normal breathing. The significance of the part played in the spread infection by each of these activities may be gauged according to the number of droplets which it produces and according to the frequency of its performance. Generally, it has been found that sneezing and coughing produce many droplets, while speaking, laughing and breathing produce few. These latter activities may, however, be of considerable importance, for their performance is frequent and, moreover, they afford the only means of droplet-spray production in the case of healthy carriers, who normally neither cough nor sneeze*…” pp. 385–386). And also: “*Thus, to assess the chances of air infection being produced by droplet-spray, information is required concerning the localities from which droplets, especially small droplets, may originate during the various expiratory activities, and also concerning the numbers of droplets which may arise from each site*….” (p. 387).

Considerable efforts have since been devoted to measuring the size distribution of the droplets emitted by expiratory events, either normal breathing and speaking or violent ejections associated with sneezing or coughing. The underlying assumption of most investigations is that the ejected droplets would form within the respiratory system, i.e. before being emitted. Different experimental techniques have been used to measure the size distribution of the exhaled droplets and, surprisingly enough, results of different investigations can differ broadly, even by orders of magnitude. Let us provide a brief overview of the picture offered by the state of the art.

The first systematic measurements presented in literature date back to the papers of Wells (1934) and Duguid (1945, 1946). These measurements were carried out sampling the droplet spray released by coughing, sneezing or simply speaking on glass or Plexiglass plates placed in front of the mouth of the subject under examination. The traces left on the plates were then analyzed under the microscope using an empirical correlation to obtain the size distribution of the original droplets. A slit impactor was instead used to collect the finest size fractions (around 1–2 μm). Alternatively, Jennison (1942) counted the photographic images of the droplets taken at high speed against a black background and enlarged. Comparable results have been obtained more recently (Xie et al. 2009) by means of a similar technique for collecting the larger droplets and an Optical Particle Counter (OPC, size range 0.3–20 μm) for directly measuring the size of smaller droplets or droplet nuclei. The size of the collected droplets was then correlated to that of the original exhaled droplets on the basis of the time spent by the droplet to fly from the mouth to the sampling position. The authors called it “residence time” and estimated it calculating the time taken by the droplet to freely fall from the mouth height to the height of the sampling position. The original size of the droplets was then calculated based on the evaporation model proposed by Xie et al. (2007) (see Sect. 3.2).

Papineni and Rosenthal (1997) used a combination of OPC and Analytical Transmission Electron Microscope (ATEM). Contrary to previous studies, these authors found a predominance of sub-micron particles (80–90%) within the exhaled droplet spray. In general, coughing produced the largest droplet concentrations and nose breathing the least, although considerable inter-subject variability was observed. One of the limitations of the measurements of Papineni and Rosenthal (1997) (pointed out by Morawska 2009) was the uncertain relationship between the size of the collected droplets and that of the originally exhaled ones. In fact, the droplets remained suspended in the air before sampling for a sufficient time to allow at least partial evaporation. It could then be hypothesized that the measured droplets were, in fact, the residues left after evaporation.

Yang et al. (2007), Morawska (2009) and Johnson (2011) used an Aerodynamic Particle Sizer (APS, size range 0.5–20 μm) to measure particle size and concentration. In particular, Yang et al. (2007) analyzed cough emissions distinguishing between droplets and residues. The droplets were collected in a sampling bag and then sized. The droplet residues were directly measured by a Scanning Mobility Particle Sizer (SMPS, size range 3 nm–1 μm). The droplet size spectra exhibited a tri-modal distribution over a size range 0.62–15.9 μm with an average value around 8.35 μm, while the size of the residues ranged between 0.58 and 5.42 μm. The results of Morawska (2009) were only in partial agreement with those of Papineni and Rosenthal (1997). In particular, the vast majority of the droplets was in the very fine range, between 0.1 and 1 μm. The particle concentration depended also on the type of emission, with maxima associated with coughing and minima to normal breathing. On the other hand, speech released a concentration of particles one order of magnitude higher than normal breathing, strongly depending on the voice loudness. In addition, the particle number concentrations measured by Morawska (2009) were over one order of magnitude higher than those measured by Papineni and Rosenthal (1997) and three orders of magnitude lower than in Yang et al. (2007), highlighting the complexity of these experiments and the influence of the specific measurement technique.

The latter important issue was tackled by Morawska (2009) reanalyzing the results of their previous experiments. Evaluating the time between particle release and measurement of particle size, they inferred that in their experiments the droplets had attained the equilibrium size resulting from the evaporation process. These results were extended by Johnson (2011) who, in addition to the APS, used Droplet Deposition Analysis (DDA) to size the droplets larger than 20 μm. This experimental set-up allowed the identification of a tri-modal distribution of the emitted droplets, with modal diameters of 1.6, 2.5 and 145 μm or 1.6, 1.7 and 123 μm in the case of speech or cough, respectively. The authors speculated that the three modes are associated with three distinct locations of droplet formation: one occurring deep in the lower respiratory tract (bronchioles), another in the region of the larynx and a third in the upper respiratory tract including the oral cavity.

The importance of sampling the droplet spray as close as possible to the mouth to avoid both the evaporation of the droplets and the dilution of the exhaled breath was recognized by Chao et al. (2009). For this reason, these authors used an interferometric technique (Interferometric Mie Imaging, IMI) that provides an accurate measurement of the droplet size. This occurs because on the non-focal plane two laser rays interfere with each other and regular fringes are observed. Their origin can be understood in terms of simple geometric theory, and their spacing can be related to the droplet diameter. This technique is suitable for transparent spherical droplets and has the advantage of allowing non-invasive measurements very close to the mouth. These authors also measured the velocity of the expiration air jet by means of Particle Image Velocimetry (PIV). Their main results can be summarized as follows:

i) the average exhalation velocity was 11.7 m/s for cough and 3.9 m/s for speech;
ii) the median diameter of the exhaled droplets was 13.5 μm for cough and 16 μm for speech;
iii) the total number of exhaled droplets was in the range 947–2085 for cough and 112–6720 for speech;
iv) the estimated evaporation of the droplets was found to be negligible.

**Fig. 8:**
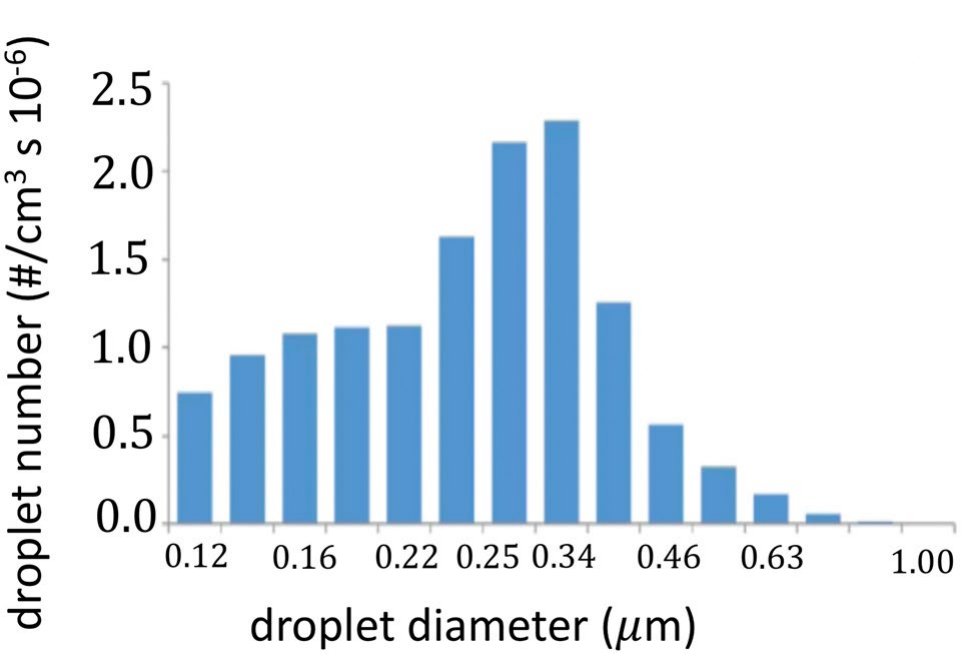
Size distribution of the droplets emitted by cough (adapted from Zayas et al. 2012)

Further non-invasive measurements were carried out by Zayas et al. (2012) and Han et al. (2013) with the help of a laser diffraction technique. In particular, the purpose of Zayas et al. (2012) was to characterize the aerosol distribution released by human cough, with the aim of developing a standard model for Influenza Pandemic control. Indeed, as discussed in Sect. 4.3, cough represents one of the main mechanisms to remove the mucus lining the human airways that is entrained by the high-speed airflow associated with the expulsive phase. The laser diffraction system allows to measure the concentration of droplets, assumed spherical, in a size range 0.1–900 μm with a sampling frequency of 2.5 s^−1^. The results, presented in Fig. 8, show that the droplets in the sub-micron range, represent 97% of the exhaled spray droplets for each single cough event.

**Fig. 9:**
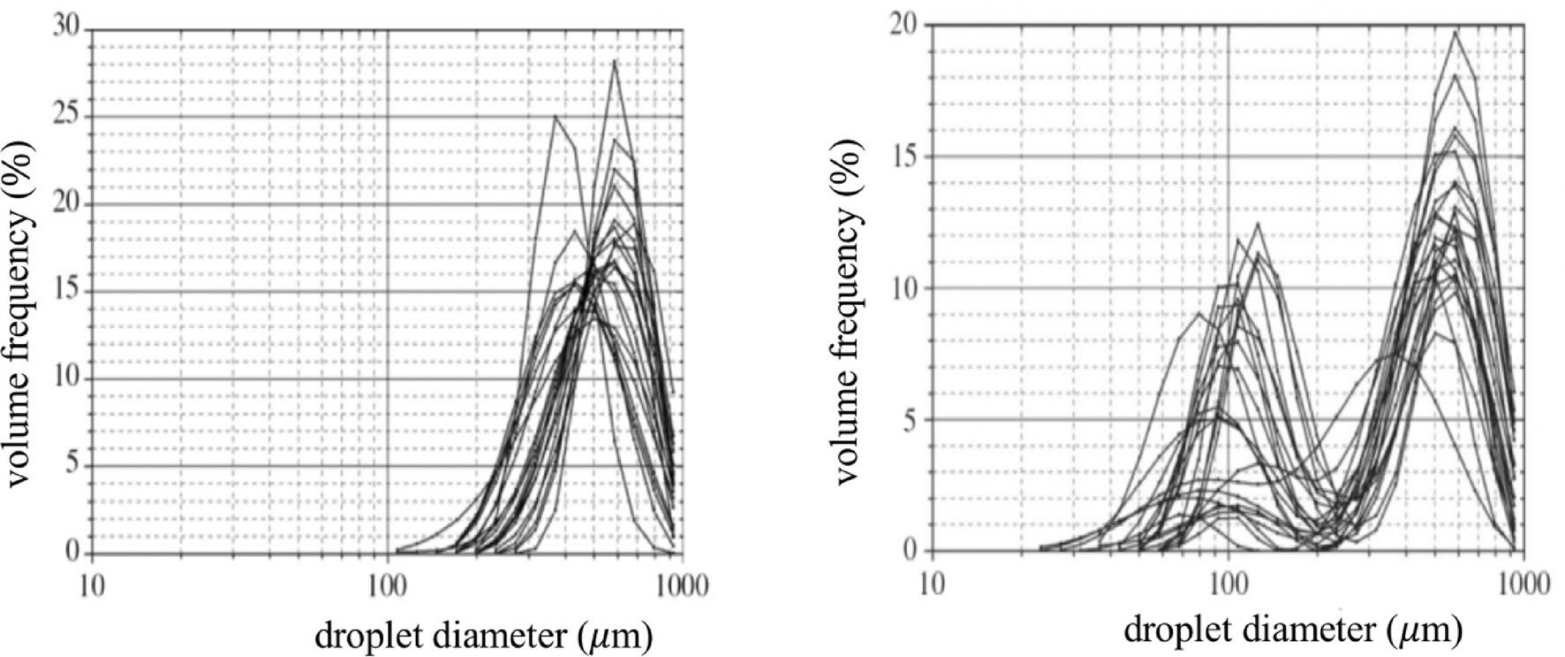
Unimodal (left) and bimodal (right) distributions of the volumes of droplets recorded for sneeze emissions of 23 patients (adapted from Han et al. 2013)

On the other hand, Han et al. (2013) focused on the more powerful respiratory events caused by sneezing. The resulting picture changes again. The first important observation concerns the type of grain size distribution. Uni-modal droplet size distributions (Fig. 9a) were found in the case of twelve patients, for ten others the distribution was bi-modal (Fig. 9b) and, in the case of other three patients, both uni- and bi-modal distributions were recorded. The size distribution was stable during the sneezing events, lasting 0.3–0.7 s*.* The second important observation concerns the average values of the size distributions: 360.1 μm in the uni-modal case and 74.4 μm in the bi-modal case (with average values for the two peaks 386.2 and 72.0 μm, respectively). These values are much higher than those measured by other authors, although for different expiratory events.

**Fig. 10:**
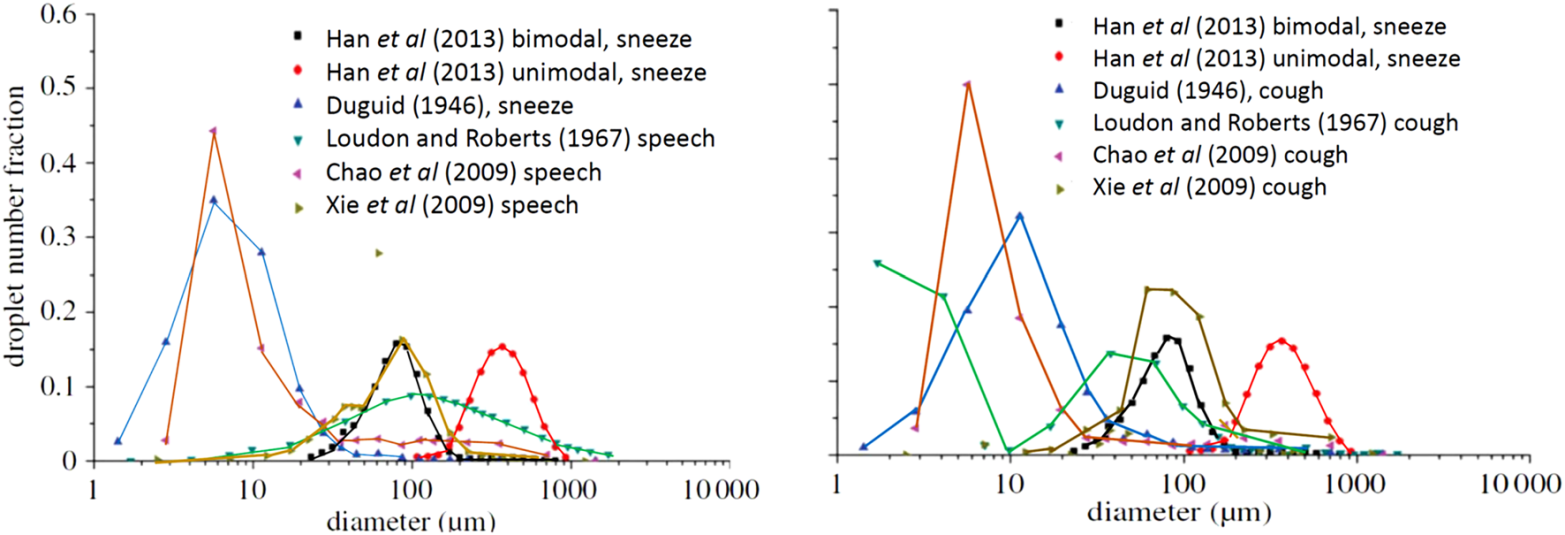
Comparison between the size distributions of the droplets emitted by sneeze and speech (left) or sneeze and cough (right) as measured by different authors (adapted from Han et al. 2013)

This is clear from a glance at Fig. 10, which shows a comparison between the results obtained by Han et al. (2013) and those of various other authors. This comparison, although far from being representative of the wealth of data reported in the literature, is sufficient to highlight the level of uncertainty that still exists on this phenomenon.

The reasons for this uncertainty are manifold. Some of them are connected to the different experimental techniques employed in different investigations. In particular, the distance between the emission source and the sizing instrumentation implies a different rate of droplet evaporation. Furthermore, the different techniques have different accuracies, although this can hardly account for the dramatic differences of results.

Two critical aspects, on which research is progressing, may help explaining the disappointing outcome of the investigations reviewed above. We need to fully understand the fluid dynamics of droplet formation and the dynamics of the evolution of the two-phase flow associated with expiratory events. These aspects are discussed in the next section.

### Experimental observations of the dynamics of expiratory events

We now investigate the physical mechanisms that control the dynamics of expiratory events. It is convenient, in this respect, to distinguish among the various typologies of such events, namely sneezing, coughing, speaking and simply breathing.

#### Coughing

**Fig. 11:**
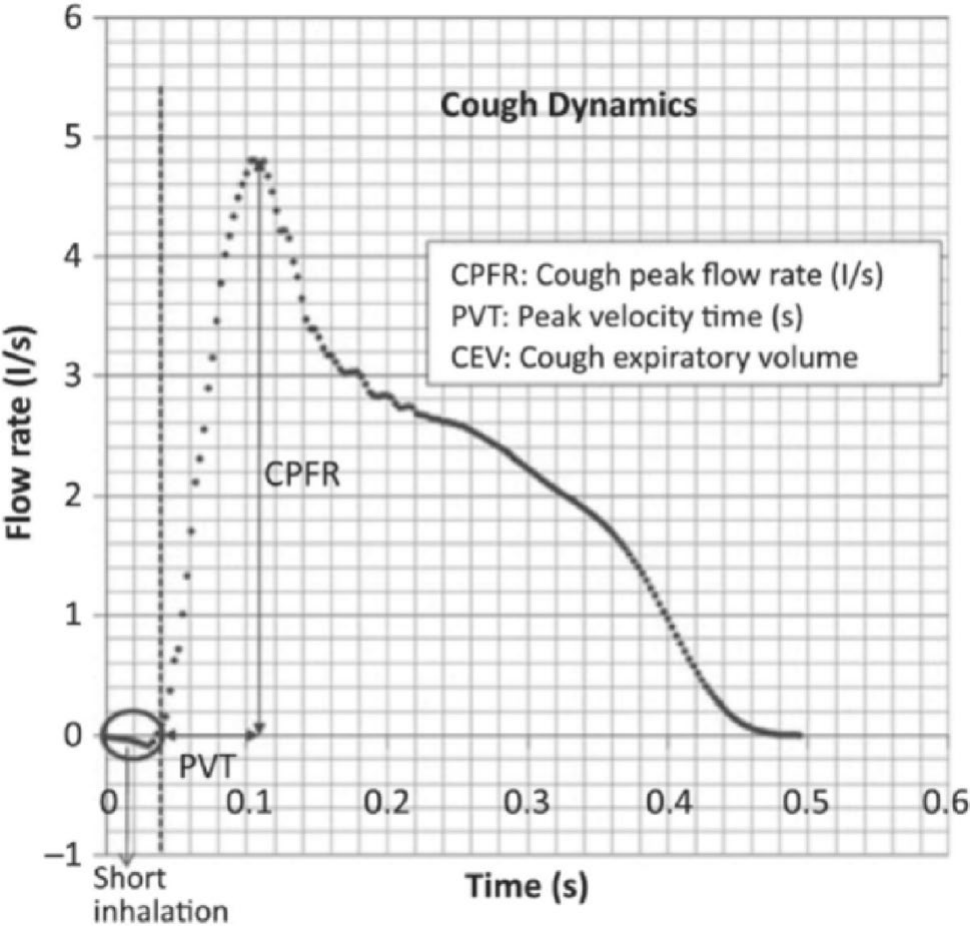
Characteristic trend of the expiratory flow rate associated with a cough event. The original reference reports the measured values of the peak flow rate (CPFR), the total expired volume (CEV) and the peak velocity time (PVT). The measured CPFR ranges are 3–8.5 (l/s) for males, and 1.6–6 l/s for females. Analogously, the CEV ranges are 400–1600 ml for males and 250–1250 ml for females; PVT: 57–96 ms (males) and 57–110 ms (females) (adapted from Gupta et al. 2009)

The integral properties of the expiratory flux associated with coughing have been widely investigated. Figure 11 shows a typical dependence of the expiratory flow rate on time, as measured by Gupta et al. (2009) for a cough event. Also shown are a few characteristic properties of the cough phenomenon. Note, in particular, the initial, short and weak inhalation which precedes expiration. The typical duration of a cough event is 200–500 ms, mouth opening of male subjects averages (4 ± 0.95) cm^2^, the Reynolds number is about 10^4^. The latter has been estimated from the peak flow rate of Fig. 11 and from the average radius of a mouth opening of 4 cm^2^*.*

Similar values have been obtained by other authors, as discussed by Gupta et al. (2009) and Bourouiba et al. (2014).

Progress in the understanding of the mechanics of the expiratory process has been recently made through visualization of the cloud typically released by distinct expiratory events. Schlieren and high-speed imaging techniques have been typically employed. A review of the early contributions is reported by Gupta et al. (2009).

**Fig. 12:**
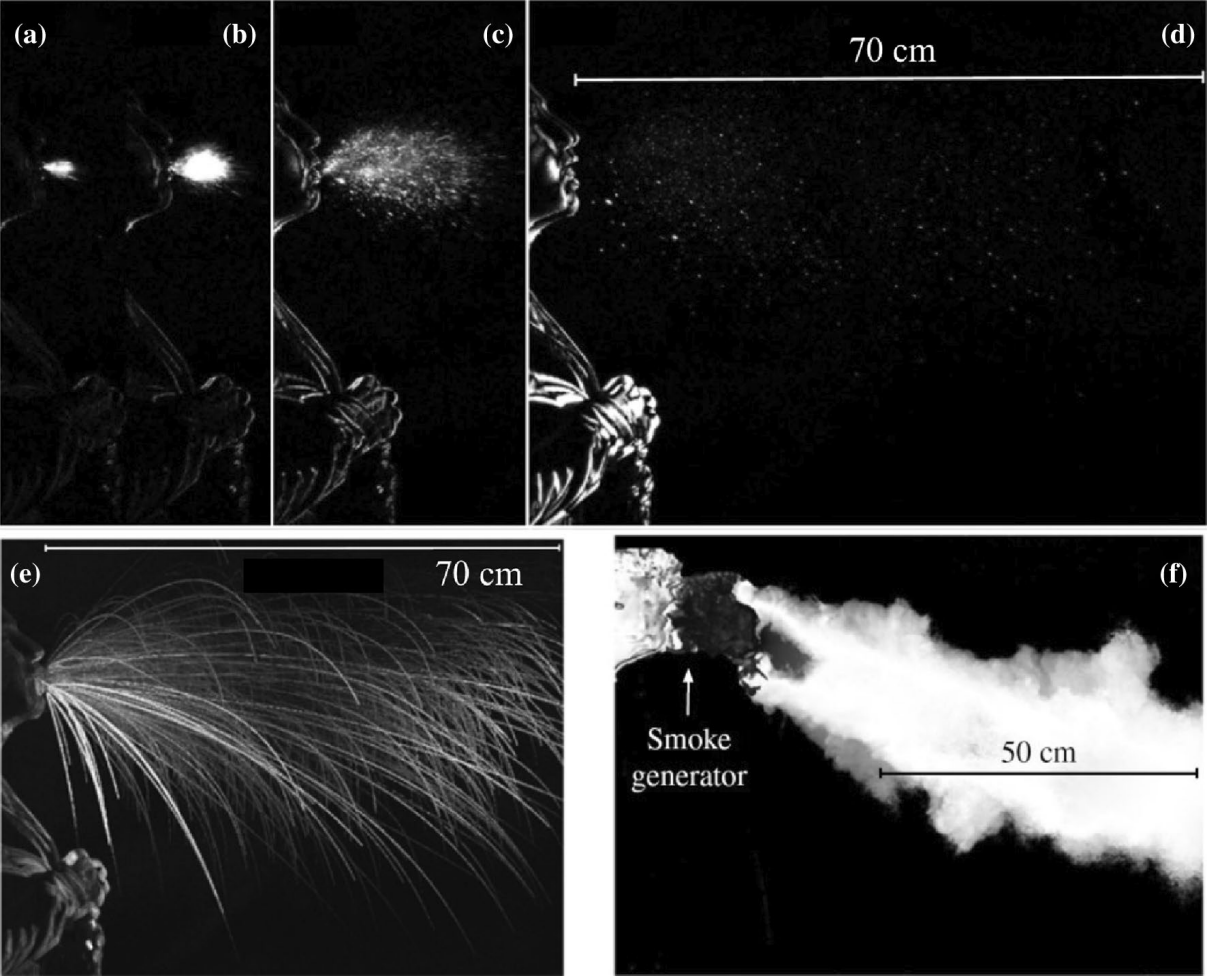
Images of the cloud released by a cough event recorded at a frequency of 1000 fps. **a** 0.006 s, **b** 0.01 s, **c** 0.029 s and **d** 0.106 s. **e** Ballistic trajectories of the largest droplets. **f** Smoke visualization of the motion of the gas phase recorded at 2000 fps. In **e** the instantaneous images of the trajectories of all the droplets recorded throughout the entire sequence are superimposed. Similarly for the smoke particles in Fig. 9f (adapted from Bourouiba et al. 2014)

The recent work of Bourouiba et al. (2014) deserves special attention. Images were recorded at a frequency of 1000–4000 frames per second (fps). Adding smoke in some experiments allowed to track the flow of the gas phase. The expiratory flux consisted of a turbulent gas cloud containing suspended droplets. The larger ones followed ballistic trajectories, which were not affected by the motion of the gas phase significantly. The smaller ones remained in suspension in the turbulent cloud and reached larger distances from the source. Figures 12a–d show results of cough visualization recorded at a frequency of 1000 fps. The sequence illustrates the evolution of the cloud up to 106 ms from the start of the expiratory event. Figures 12a–c suggest that, in the initial phase, the cloud has a cone shape and the droplet concentration is high. Figure 12e shows the ballistic trajectories of the largest droplets. Figure 12f shows the smoke visualization of the motion of the gas phase, recorded at a 2000 fps.

It is well known that flows of homogeneous fluids issuing from localized sources can be classified as jets, puffs, plumes and thermals. Jets and plumes are both generated by persistent sources but they differ as the driving force of the former flows is momentum whilst the latter flows are driven by buoyancy, i.e. the excess (or defect) of gravity due to the different density of the cloud relative to the environment. Puffs and thermals are the equivalent of jets and plumes when the source is not persistent. A puff represents the final stage of jet evolution once the momentum injection from the source stops.

It is also well known that a common characteristic of all these flows is the process of entrainment, whereby the cloud mixes with the air of the surrounding environment. As the latter is initially still, the cloud slows down as it moves away from the source, an effect displayed by the divergence of the cloud. The cough cloud exhibits a mixed behavior. In the initial (expulsion) phase the jet behavior dominates. Indeed, buoyancy, that is driven by the difference between the cloud temperature and the temperature of the ambient air, is negligible in this phase. At the end of the expulsion phase, the jet evolves into a puff. Moreover, the puff is progressively affected by buoyancy as the cloud loses momentum, hence a puff-thermal behavior eventually emerges. It is buoyancy that controls the curvature of the cloud trajectory whereby the cloud, which is initially inclined downward by an angle of 24 ± 7° with respect to the horizontal (Bourouiba et al. 2014), tends to rise upward in the far field.

The process is further complicated by the fact that the released cloud may hardly be interpreted as a homogeneous fluid. It rather consists of a two-phase mixture of droplets dispersed into a fluid phase which is hotter and more humid than the ambient air. This has the important consequence that the mixture characteristics change as it moves away from the source. On the one hand, as already pointed out, larger droplets settle. On the other hand, smaller droplets are carried by the cloud and undergo evaporation depending on the variation of the temperature and relative humidity fields. We will return to these features in the next section, where we will outline some known attempts to model the process and discuss the issues that still await to be fully explored.

#### Sneezing

**Fig. 13:**
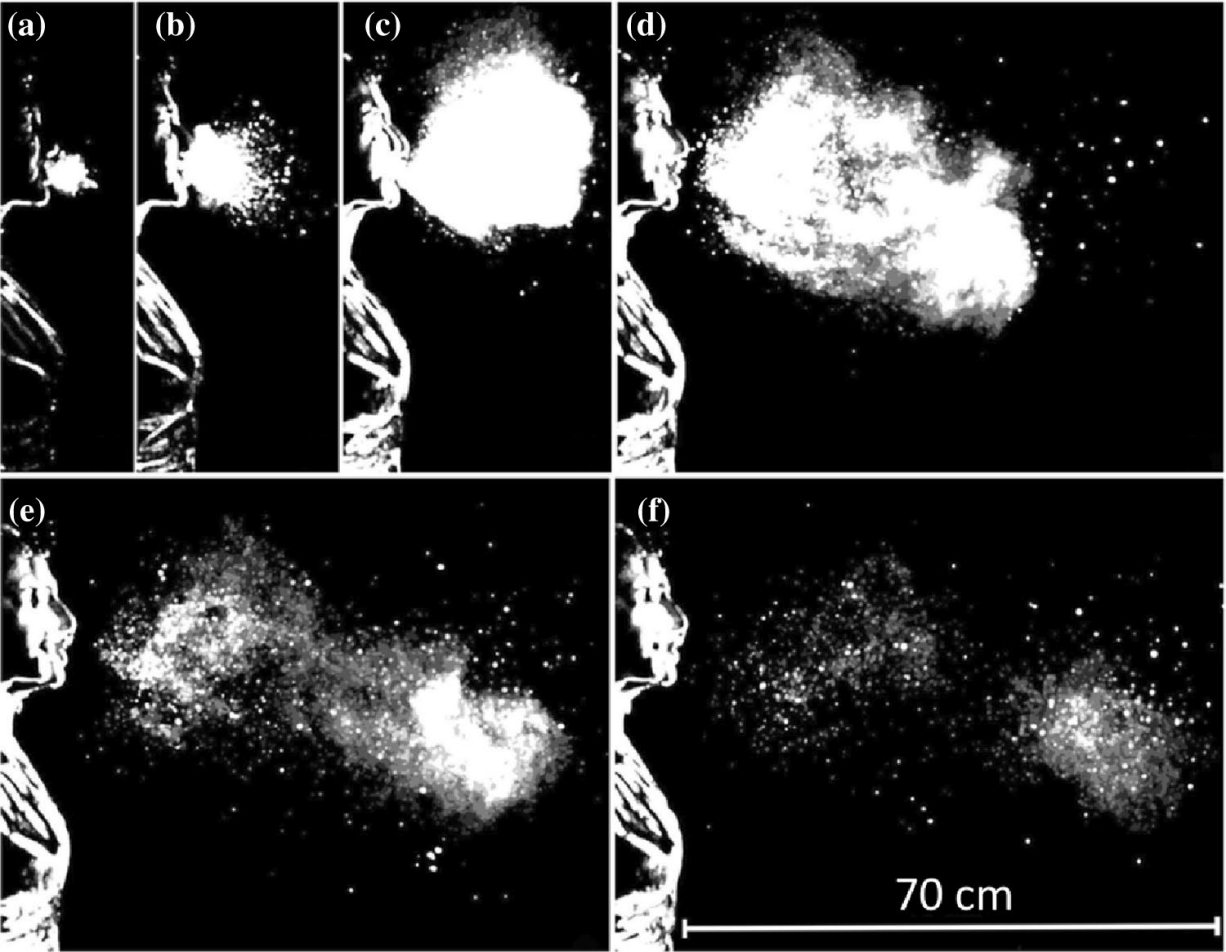
Images of the cloud expelled by sneezing, recorded at a frequency of 1000 fps **a** 0.007 s, **b** 0.03 s, **c** 0.107 s, **d** 0.162 s, **e** 0.251 s, **f** 0.34 s (adapted from Borouiba et al. 2014)

The above observations have shown that, in the cough case, the liquid component of the cloud consists of droplets already in an immediate neighborhood of the mouth. The case of sneezing turns out to be more complicated. Figure 13 shows a sequence of images of the structure of the cloud expelled by a sneeze (Bourouiba et al. 2014), visualized by a technique identical with that employed for coughs. The duration of the event was 200–250 ms*.* The initial value of the Reynolds number was estimated at 4 × 10^4^, i.e. the strength of the sneeze expulsion was roughly four times larger than cough. Other features of the cloud were similar to those found in the cough case, notably the loss of large droplets settling in the initial phase and the onset of buoyancy effects, which let the cloud trajectory deviate upward. However, a distinct feature of the sneeze cloud was its large density in the initial phase. At this stage, the liquid component of the cloud did not consist of droplets, but rather of structures of fairly large size, which were still discernible at some distance from the mouth.

**Fig. 14:**
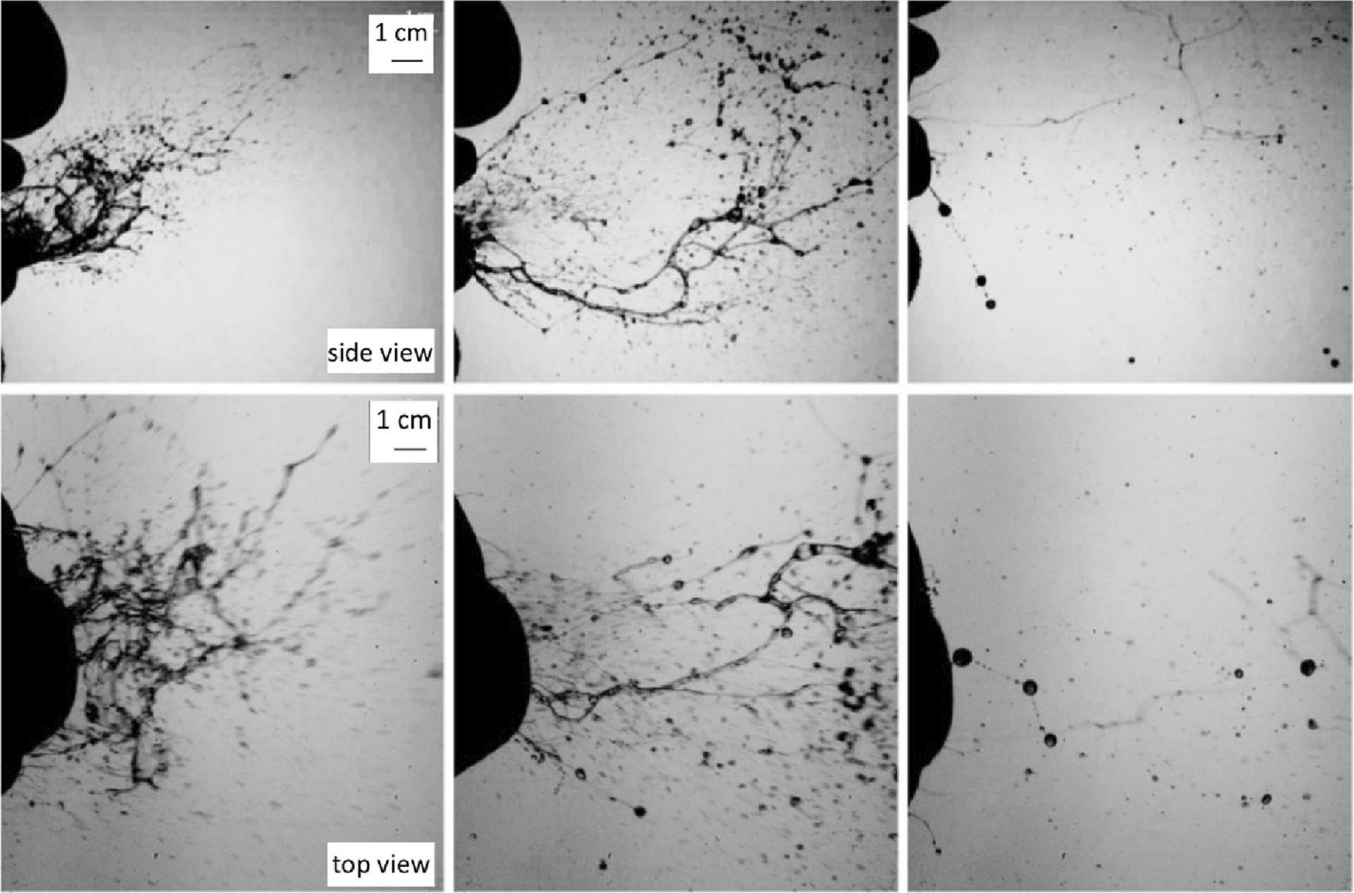
Lateral (upper panel, 8000 fps) and top (lower panel, 2000 fps) views of the initial phase of the expiratory expulsion associated with a sneeze. Droplets (right column, *t* = 117 ms) form from the fragmentation of complex structures evolving from sheets and bags (left column, *t* = 8 ms) into elongated filaments (central column, *t* = 21 ms) (adapted from Scharfman et al. 2016)

This feature may bear some relevance to the issue of understanding the large differences between the size distributions of droplets measured by different Authors. It was further investigated by Scharfman et al. (2016) who used the same technique of Bourouiba et al. (2014) with larger sampling frequencies (8000 fps). Scharfman et al. (2016) observations (Fig. 14) show clearly that, in the case of sneezes, the fragmentation processes leading to droplet formation persist after the expiratory ejection. Indeed, Fig. 14 shows the presence of actual droplets only after 117 ms (left column), and their formation appears to result from the fragmentation of more complex structures that, in the expulsion phase consist of sheets and bags (left column, *t* = 8 ms). These then evolved into elongated structures in the form of filaments displaying the presence of pearls (central column, *t* = 21 ms).

#### Speech

**Fig. 15:**
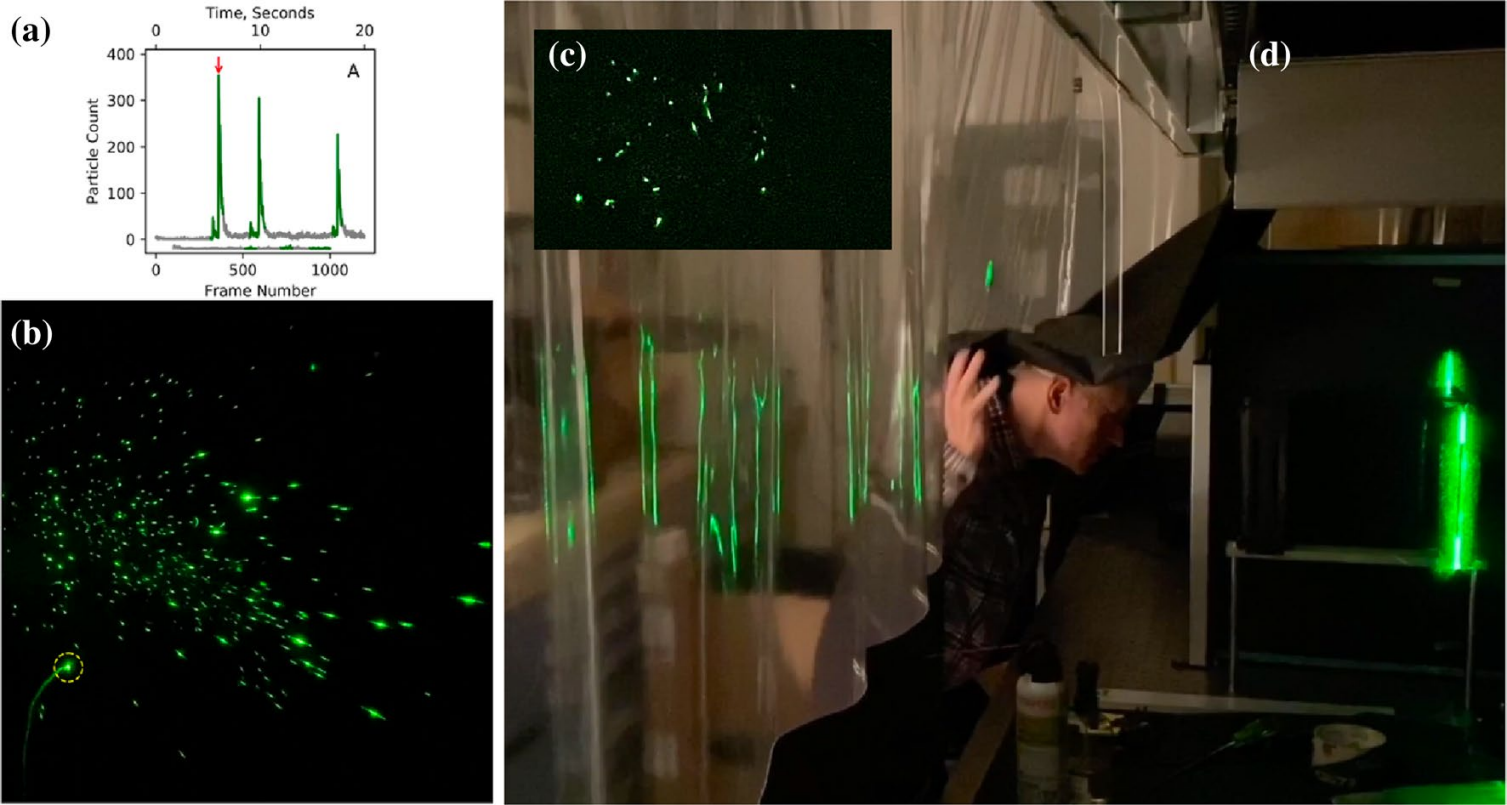
Panel A shows the number of flashes that were recorded in a single video frame. Sampling frequency was 60 fps. Green denotes the time when the person spoke. Note that, during the silent intervals (grey line), the number of flashes did not vanish immediately, presumably because a few droplets remained in the light sheet for a few seconds after speaking stopped (Anfinrud et al. 2020). **b** Shows a photogram corresponding to a peak in droplets emission (see arrow in **a**). The different brightness of individual flashes indicates the different droplet size (images provided by Adriaan Bax). Panel C shows a snapshot of saliva droplets, and Panel D an image of the experimental setup (photo credit: National Institute of Diabetes and Digestive and Kidney Diseases, National Institutes of Health)

Less attention has been devoted so far to investigate the expiratory events associated with normal or loud speaking. Recently Anfinrud et al. (2020) have reported results of an experiment where the cloud released by an individual who repeated many times the same sentence (‘stay healthy’) was visualized. The technique consisted of the generation of a vertical laser sheet, 1 mm thick and 15 cm high, which was directed through slits on opposite sides of a cardboard box whose interior was painted black. When the individual pronounced a word, a cloud of droplets was released. Droplets moved along a distance of 50–75 mm before they crossed the laser sheet. As a droplet crossed the sheet, a flash was produced (Fig. 15b). Its brightness was a function of the particle size as well as of the fraction of time the particle remained on a single video frame. Sampling frequency was 60 *fps*. The original video can be downloaded from the following site: https://doi.org/10.5281/zenodo.3770559).

Figure 15 A shows that the number of flashes recorded in a single frame has a peak (see arrow). The peak was found to be clearly associated with the pronunciation of the letters “th” of the word “healthy”. Repeating the same sentence three times, with short intervals between them, gave rise to similar emissions, with peak values of the number of flashes depending on how loud was the speech. The light scattering method proves extremely sensitive, i.e. it allows to reveal the presence of medium-sized (10–100 μm) droplets, which remain in suspension for at least 30 s. As a result, the estimated values of the average droplet emission rates were 2600 s^−1^ with peaks as high as 10,000 s^−1^, values much larger than those detected in previous works of Duguid (1946), Morawska (2009), Chao et al. (2009) and Asadi et al. (2019)

More recently, the same research group (Stadnytskyi et al. 2020) derived quantitative estimates for both the number and size of the droplets that remain airborne. Essentially, these authors used an internal fan to mix the cloud inside the box, turned it off 10 s after the speech was terminated, and kept recording for as long as 80 min. Analyzing the movie clip frame by frame, they then observed an exponential decay of the number of scattering particles, from which they estimated a half-life in the enclosure of ca. 8 min. Assuming that the latter corresponds to the settling time of a particle in the box, they estimated that the droplet nucleus had a size of roughly 4 μm. At the relative humidity and temperature of the experiment, the dehydrated particle of 4 μm corresponded roughly to a hydrated droplet of ca. 12- to 21-μm size. At an average viral load of 7 × 10^6^ virions per milliliter (Wölfel et al. 2020, cfr Sect. 3.1), Stadnytskyi et al. (2020) conclude that “… 1 min of loud speaking generates at least 1000 virion-containing droplet nuclei that remain airborne for more than 8 min”. These estimates assume an average value of the viral load, which is known (cfr Sect. 3.1) to vary significantly, reaching peaks more than two orders of magnitude larger than the average. Hence, the number of virion-containing droplet nuclei released by loud speaking may be much larger than the above estimate. Moreover, the latter is conservative as the visualization technique was unable to detect the smallest fraction of the emitted droplets.

### A short digression: where and how droplets form

We have ignored so far an aspect that affects the dynamics of the expiratory cloud only indirectly: where and how the cloud droplets form. This is a conceptually important issue, which poses a number of open fluid dynamics problems.

Droplet formation arises from a number of processes dependent on a variety of factors. Firstly, the type of expiratory event: indeed, in the previous section, we have seen that different expiratory events (cough, sneeze, speech), are characterized by flow velocities and cloud composition that vary significantly. Secondly, the nature of internal boundaries, which may be moving boundaries. Thirdly, the interaction between the various fluids that are present in the respiratory system, play a crucial role. Indeed, as pointed out in Sect. 2.2, the human airways, in the first 15 or so branches, are coated with a double liquid layer, with an outer mucus blanket superimposed on an inner serum layer (Fig. 3). Serum is a Newtonian fluid, whilst mucus is a complex material with viscoelastic properties, a yield stress and thixotropic behavior (e.g. Powell et al. 1974). The non-Newtonian rheology of physiological fluids is a serious obstacle to the modelling of droplet formation via suitable numerical methods. This difficulty adds up to other difficulties already seen in relation to the numerical modelling of turbulence in the jet/puff stage and to provide a consistent description of the whole stage of the droplet evaporation process (e.g. Wei and Li 2015, 2017).

The thickness of the double layer is typically of the order of 5–10 μm in the larger airways. The liquid layer has a protective lubrication effect for the underlying cells as well as a trapping function for inhaled particles and dangerous microbes. The process of removal of the mucus layer plays a major defensive role for the lungs. Various mechanisms contribute to this process, some of them directly relevant for the generation of droplets expelled by expiratory events.

Under ordinary conditions, two main mechanisms, namely gravitational clearance and ciliary propulsion, operate. In particular, ciliary propulsion has been the subject of several investigations starting from the early works of Blake (1971, 1975) and Ross and Corrsin (1974). These works are reviewed by Grotberg (1994). Essentially, each cilium beats with typical frequency around 1.5 Hz and its tip moves following an elliptical trajectory. Beating of different cilia are coordinated, such that their overall behavior leads to a wave perturbation, which propagates away from the mouth with wavelength of 30 μm, and drives mucus transport with a velocity of 0.2 mm/s. The above physics justifies the framework used in some analytical models of ciliary propulsion where cilia are modeled as a continuous wall subject to the propagation of a traveling wave. Mucus clearance driven by ciliary propulsion is clearly a fairly slow process, which does not bear a direct relevance to the subject of the present review.

**Fig. 16:**
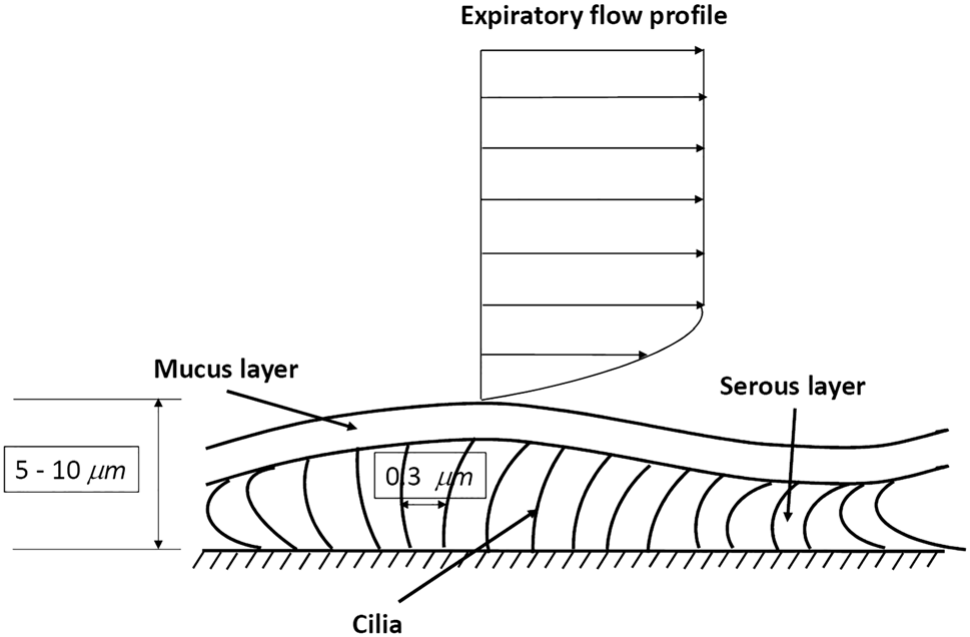
Sketch illustrating the double liquid layer coating the human airways (adapted from Grotberg 1994)

Droplet formation is associated with more violent events. Let us first note that, under normal conditions, the air speed in the trachea (an airway with a diameter of 14 mm) is about 6.5 m/s (Ross et al. 1955), with peak measured value of the flow rate of 1 l/s. When a person coughs these values increase considerably: the peak flow rate reaches values above 7 l/s. With the same area of the cross section of trachea, this would imply air speeds around 46.5 m/s. These values increase further the need for taking into account that coughing is associated with the collapse of trachea with a consequent reduction of its diameter, which nearly halves. This would suggest that the air speed might reach peaks higher than 200 m/s and Reynolds numbers around 2 10^5^ that would definitely be associated with a turbulent character of the air flow (Ross et al. 1955) (Fig. 16).

An air flow characterized by speeds of the order of tens of m/s is potentially able to destabilize the air–mucus interface through the well-known mechanism of Kelvin–Helmholtz hydrodynamic instability. Essentially, the air flow generates a shear at the air–mucus interface which is sufficiently intense as to allow the growth of essentially inviscid perturbations in the form of interfacial waves. Their amplitude can grow so much that the destabilized mucus undergoes a process of fragmentation and atomization into droplets, just like in breaking sea waves.

Only the initial phase of this complex process has been investigated so far. In particular, Moriarty and Grotberg (1999) performed a linear stability analysis of the motion of the double layer subject to an air flow and showed that instability occurs for values of the air speed strongly dependent on the value of the surface tension *σ* at the air–mucus interface. The critical speed is about 5 m*/s* for a value of *σ* equal to 10 dyn/cm. Needless to say, a linear stability analysis is unable to predict the structure of perturbations as they attain finite amplitudes. To achieve this goal, and then analyze the fragmentation process leading to droplet formation, numerical solutions of the fully nonlinear problem are needed. This is not an easy task, as fragmentation is associated with changes of the interface topology and the development of cusps when two interfaces reconnect (see the review of Scardovelli and Zaleski 1999). This is an actively investigated area of research due to its relevance for many engineering applications, which will likely take advantage of the continuous increase of the computational power (Wang et al. 2016).

**Fig. 17:**
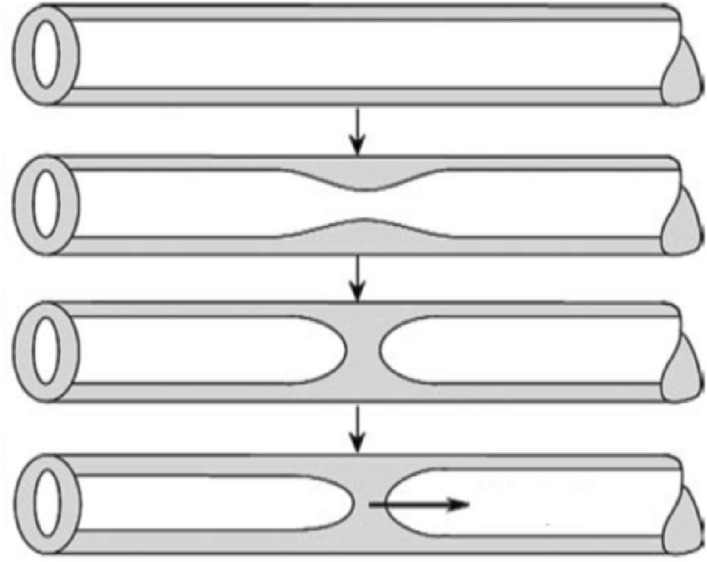
Sketch illustrating the mechanism whereby the instability of the mucus layer lining an airway may lead to its occlusion (adapted from Malashenko et al. 2009)

Kelvin–Helmoltz instability is not the only mechanism that can lead to the fragmentation of the mucus layer. As we will see in the following, a second instability, the so-called Plateau–Rayleigh instability, may play an important role in the human airways. This instability explains the experimental observation (Plateau 1873) that a sufficiently long vertically falling stream of water breaks up into drops. Rayleigh (1879) showed that instability arises from the effect of surface tension at the air–liquid interface. Neglecting viscous effects, the wavelength of the most unstable perturbations turn out to be equal to the circumference of the falling water column. A similar mechanism may occur in the human airways: a small perturbation of the air–mucus interface may grow enough to let the interface reach the axis of the cylindrical conduit (Fig. 17). Under these conditions, the airway is occluded (Romanò et al. 2019) and the occlusion may propagate until it disintegrates into small droplets (Malashenko et al. 2009).

A further mechanism suggested by Malashenko et al. (2009) and Almstrand et al. (2010) is related to the rupture of menisci formed by the respiratory tract lining fluid at the level of the terminal bronchioles. These conduits have the size of the order of a millimeter, and it is known that their closure typically occurs during a progressive slow exhalation. The reopening of these peripheral airways during the following inspiration is thought to produce droplets of micron size due to the rupture of menisci. Modeling this process is a further open problem of difficult solution.

Finally, as discussed in Sect. 3.2, the experimental observations of Scharfman et al. (2016) suggest that violent respiratory events generate clouds containing extended liquid structures (bags, sheets, filaments) whose fragmentation gives rise to droplet formation through a variety of mechanisms reviewed by Villermaux (2007). Modeling the fragmentation process to predict the size distribution of the resulting droplets represents an open challenge for future research.

### Open problems in the fluid dynamics of the two-phase flow generated by expiratory events

The scenario arising from the experimental observations discussed in Sect. 4.2 opens to new challenging questions that still await adequate answers.

The implementation of predictive models for the dynamics of the exhaled air emitted through respiratory events is of paramount importance for a deep comprehension of the long-range transport mechanisms responsible for the infection spread far from the emission source.

Some attempts in this direction appeared in the literature. In way of example, Bourouiba et al. (2014) provided a simple interpretation of their experimental results obtained in the laboratory by generating a two-phase flow consisting of freshwater with heavier particles dispersed in it, which was abruptly introduced, through a piston, into a tank containing salty (and, therefore, denser) water. The experiments mimics a cloud of warmer (with respect to the ambient) air hosting droplets as it happens during a respiratory emission. We mention in particular: the initial jet behavior of the emitted fresh fluid, its transformation into a puff-thermal in the far field, and, finally, the evolution of the two-phase mixture induced both by the entrainment process and by the sedimentation of the transported particles. The interpretation of Bourouiba et al. (2014) is based on a simple model which is, however, worth discussing.

The cloud is treated as a volume that evolves while maintaining its self-similarity. Indicating by *r* the characteristic 'radius' of the cloud, the entrainment is described assuming that *r* = *α s*(*t*) with *α* being the entrainment coefficient estimated from the experimental data and *s*(*t*) is the longitudinal coordinate of the cloud center of mass, defined along its trajectory. The initial jet behavior is therefore accounted for by imposing the conservation of the momentum flux *M*_0_ ~ *ρ* *r*^2^ (d*s/*d*t*)^2^, a condition which, together with self-similarity, easily leads to the power-law behavior *s*(*t*) ~ *t*^1/2^. The far field puff behavior is described by imposing the constancy of the momentum of the cloud *I*_0_ ~ * ρ r*^3^ (d*s/*d*t*), which, exploiting the self-similarity, leads to the dependence: *s(t)* ~ *t*^1/4^. This result coincides with the power-law behavior originally obtained by Kovasznay et al. (1973).

**Fig. 18:**
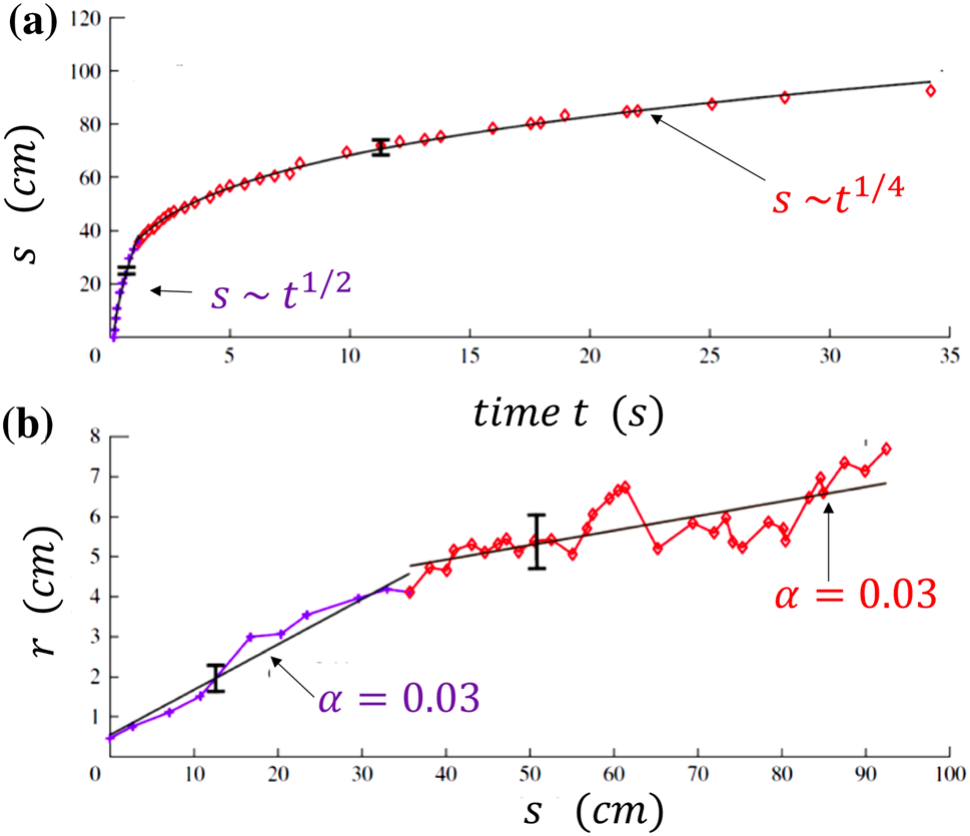
Results of experimental observations on the trajectory of the cloud of exhaled air and its characteristic size compared with the predictions of a simple theoretical model. Here, *r* is the characteristic radius of the cloud (cm), and *s* (cm) is the longitudinal coordinate of the cloud’s center of mass, defined along its trajectory. **a** Time evolution of *s*. The two asymptotic regimes are shown; **b** The entrainment is described by assuming that* r* = α *s*(*t*) with α being a suitable entrainment coefficient estimated from the experimental data. Clearly, the results from the theoretical model depend on the choice of α (adapted from Bourouiba et al. 2014)

Figure 18a shows that the theoretical model accurately predicts the observed time dependence of *s* by estimating the entrainment coefficients from the corresponding dependence of *r* from *s* shown in Fig. 18b. The change in slope in the relation between *r* and *s* indicates the transition from the jet to the puff regime.

The previous model ignores both the buoyancy effects and the multiphase nature of the flow. Bourouiba et al. (2014) proposed a further improvement maintaining the same hypothesis of self-similarity but accounting for the buoyancy effect. This implies a progressive reduction of the cloud momentum. The latter reduction is taken into account during the evolution of the cloud only via particle sedimentation which is described in terms of a simple model because of the lack of detailed information on the carrier flow. Evaporation, a further source of momentum reduction, is not accounted for in the model. The main conclusion of this work is summarized in the following statement: drops of size smaller than 50 μm remain suspended in the cloud long enough to reach heights (4–6 m) that affect the ventilation ducts.

The limitations of this model (and its conclusions) are clear. It basically ignores two important aspects of the phenomenon: the turbulent character of the fluid motion and its two-phase nature. As we have seen, the flow associated with respiratory emissions is indeed characterized by high values (~ 10^4^) of the Reynolds number, the dimensionless parameter controlling the ability of a moving fluid to create fluctuations in its velocity field. These fluctuations characterize the turbulent character of the dynamic state of a fluid flow (Frisch 1995). Because of turbulence, the flow out of the mouth is extremely irregular, fluctuating, both in space and in time. Moreover, the mechanism of entrainment not only affects the buoyancy of the air cloud but also induces a reduction of its water vapor content. The ambient humidity is indeed smaller than the humidity of the exhaled air.

It is worth emphasizing that we will assume that the environment is not saturated (i.e. its vapor pressure is smaller than the saturated vapor pressure) and that it has, initially, a temperature lower than the exhaled air from the mouth. The hypothesis that the environment is not saturated, in cases where the temperature of the cloud (about 30–35 °C during expulsion) is comparable with that of the environment (for example about 25 °C), is equivalent to saying that the absolute humidity of the environment is sufficiently lower than that of the exhaled air. As time runs, due to the mixing of the two air masses, the temperature of the exhaled air is lowered and its absolute humidity is reduced. The first effect, by lowering the saturated vapor pressure, favors condensation, but it is immediately counteracted by the second, which favors the evaporation of the droplets.

Let us now move back to turbulence as this feature characterizes also the mixing process which determines how the exhaled air, initially saturated of water vapor, dilutes with ambient air. This mechanism is intimately chaotic characterized by persistent fluctuations in the relative humidity field. This quantity certainly decays with time but its spatial structure in the decaying process is highly nonuniform. Strong fluctuations with respect to the average relative humidity are present, a fact that justifies the term passive scalar turbulence to characterize the mixing process of the humidity field (Shraiman and Siggia 2000).

The turbulent nature of the relative humidity field can have a dramatic effect on the fate of the evolution of saliva droplets. Cloud formation in the high atmosphere provides a large-scale example of the condensation/evaporation processes taking place in the air cloud exhaled from the mouth during coughing/sneezing/talking. The crucial role of turbulent fluctuations in the relative humidity field was isolated in 2005 in relation to its role in the cloud droplet growth by condensation (Celani et al. 2005). As a result of this study, turbulence turned out to be the key ingredient to explain the observed spectrum broadening of cloud droplets resulting at the end of the condensation stage. Due to this process of size broadening, the droplets can reach different terminal velocities, a fact that allows them to start the second phase of their growth dominated by collision and coalescence.

Roughly speaking, a population of droplets all animated by the same terminal velocity would not allow the triggering of collisions and, therefore, there would be no way to form a raindrop.

For the saliva droplets, the growth by condensation is certainly not the key phenomenon at play, at least on average. On the contrary, the expelled droplets, in general, move in an under-saturated medium. In this framework, the interesting questions, still largely unanswered, concern the way and the rate at which these droplets evaporate. As we have seen in Sect. 3.2, the evaluation of these characteristics is, to date, done via mean-field arguments, which either ignore the effects of turbulent fluctuations or describe them in an extremely simplified way (see for example Liu et al. 2017). On the contrary, by reversing the way of reasoning followed to understand what happens in a cloud in the upper atmosphere, one can easily imagine that the role of turbulence is very important to determine the fate of expiratory droplets during their evaporation stage. Droplets that remain longer in less under-saturated zones will evaporate slower than other droplets remaining in regions where the relative humidity is lower.

The consequence of this way of reasoning is that the sizes of the expiratory droplets are expected to diversify before they evaporate completely, more importantly than in the absence of turbulent fluctuations in the humidity field. Since, as already seen in Sect. 3.2, the droplet sedimentation velocities are proportional to the square of their radii, the turbulent fluctuations responsible for the possible spectrum broadening of droplet sizes are expected to cause an analogous broadening of the spectrum of the droplet falling velocities in the ambient still fluid. This would imply the appearance of droplets settling abnormally faster/slower with respect to the mean-field theory predictions. Cloud droplets, evaporating faster, reach faster the stage where they have reduced to their nuclei. They are thus expected to remain airborne for longer times, which increases the probability of long-range infection. On the other hand, cloud droplets evaporating slower will maintain their inertia longer, thus experiencing a less effective ability to follow the air flow. This mechanism works to reduce the long-range virus transmission. It is currently impossible to anticipate which mechanism among the two will dominate the long-range infection transmission. Only studies dedicated to this issue will provide the final answer.

As we have anticipated in Sect. 3.2, the consequence of the above considerations crucially depend on the answer to the following fundamental question: does the infective capacity of a droplet reduced to a dry nucleus remain unchanged? And for how long? Achieving a deeper understanding of the role of turbulence in this process, dictating the fate of the droplet evaporation phase, is an issue of paramount importance. Understanding the process in detail, on a quantitative basis, is indeed a crucial prerequisite for the formulation of more realistic predictions, in relation to issues of social distancing and strategies of reduction of airborne virus transmission. A continuous interaction between experts of fluid dynamics and virologists is also a fundamental need.

### Role of natural and forced ventilation

The air ejection mechanisms analyzed in the previous sections assumed a quiet environment with assigned thermodynamic properties (temperature and relative humidity for example). The topic we are now dealing with concerns the possible role of the environment on the spread of the infection in the presence of natural (Linden 1999), or forced ventilation.

To estimate the probability of airborne transmission of an infectious agent in closed environments, subject to air changes, Riley et al (1978) developed what is now commonly called the Wells and Riley equation. Without making explicit reference to the details of the analysis, this equation is a quantitative and rational formulation of what the intuition suggests. To reduce the probability of contagion it is convenient to stay as little as possible in an environment where there are infected subjects. Moreover, a reduction of the concentration of virus in the ambient air must be sought through an appreciable exchange of the ambient air.

By adding information on (i) the emission rate of infected doses (*q*, expressed in quanta/s) injected into the air; and (ii) the pulmonary ventilation required for each susceptible subject, expressed as volumetric flow rate, the formula is able to predict the number of new infections. Note that a quantum is defined as a dose of infection such that a susceptible exposed to it has a 63% probability of actually being infected (Rudnick and Milton 2003). In deriving their equation, Wells and Riley made two fundamental assumptions: (1) the environment is well mixed and, (2) in steady-state conditions. The first hypothesis implies that an infected particle has the same probability of being anywhere in the airspace of a building, regardless of when and where it was generated.

Rudnick and Milton (2003) proposed a mathematical model that, using CO_2_ concentration as a biomarker of expelled air, does not require the assumption of steady-state conditions. The model assumes that the elimination of infective particles caused by filtration, sedimentation and other mechanisms is small compared with their removal by ventilation effects. Rudnick and Milton's model (2003) allows a more accurate prediction of the risk of infection in modern buildings where, for the sake of design and because of utility reasons, ventilation by outside air varies over time and often its flow cannot be accurately measured. The equation also allows for risk estimation in buildings and other indoor environments with poor air exchange from outside. The analysis presented by the authors shows that increasing air exchange can prevent airborne transmission of some common respiratory infections and influenza, but has a limited impact on highly contagious diseases such as measles.

The stochastic generalization of Wells and Riley's model proposed by Noakes and Sleigh (2009) overcomes the limitations associated with the hypothesis (1) made in the original model, namely the well-mixed condition. Indeed, this hypothesis is rarely verified even in indoor areas equipped with professional ventilation systems. It is not compatible with the spatial proximity that may exist between susceptible and infected people. In particular, the lack of mixing among different areas of a building affects the risk of infection in a space consisting of communicating rooms, such as hospital wards.

These effects can be accounted for using computational fluid dynamics (CFD) techniques to simulate airflow and contaminant dispersion. The outcome of simulations allows one to identify regions of good and bad mixing and areas with high contaminant concentrations that would cause a higher risk to room occupants. An alternative approach is to use the so-called zonal or network ventilation systems, capable of estimating ventilation flows in large multi-connected spaces as whole buildings. Although such models are not able to resolve local details of air flows, they have proven useful in predicting air flows and contaminant transport in a wide range of applications, including natural ventilation (Asfour and Gadi 2007).

Among the most comprehensive reviews investigating the link between ventilation in buildings and airborne infection transmission, the review by Li et al. (2007) is worth summarizing.

The authors selected the 40 best studies, based on quantitative analysis criteria, and set up a review committee composed of medical and engineering experts in the fields of microbiology, medicine, epidemiology, indoor air quality and building ventilation. Most of the members of the committee had experience in research on the 2003 SARS infection. The Committee systematically evaluated 40 original studies through both individual and joint evaluations. Ten of the 40 studies examined were considered conclusive in relation to the association between ventilation in buildings and airborne transmission of infection. According to the authors, there is substantial evidence demonstrating the association between ventilation, air flow in buildings and transmission/diffusion of infectious diseases such as tuberculosis, chickenpox, influenza, smallpox and SARS. On the contrary, again according to the authors, there are insufficient data to specify and quantify the minimum ventilation requirements in hospitals, schools, offices, houses and isolation rooms in relation to the spread of infectious diseases in air.

Ventilation systems are not only able to guarantee the exchange of air from outside but they can also change the relative humidity in the room. As far as the effect of this environmental parameter on the viral transmission is concerned, combined with that of the ambient temperature, the literature does not draw firm conclusions (Wen et al 2020). Environmental conditions corresponding to low relative humidity associated with low temperature seem to favor the stability and transmission of certain influenza viruses such as respiratory syncytial virus, human rhinovirus and avian influenza virus (Derby et al 2017; Davis et al 2016; Ikäheimo et al. 2016). On the contrary, it has been observed that for dust mite allergens and other virus types (Derby et al. 2017; Morawska et al. 2006; Weber and Stilianakis 2008) such environmental conditions appear unfavorable to the spreading of the infection.

In summary, the existing literature stresses the importance of indoor ventilation but does not allow firm conclusions on its role valid for each type of virus. It, therefore, seems appropriate to continue collecting data in the field and developing increasingly sophisticated fluid-dynamic and infection models. Their ultimate goal is to identify the minimum requirements needed to define ventilation standards in hospitals, schools, offices, homes and isolation rooms that allow to minimize, in a sustainable way, the airborne spread of infectious diseases. Viral RNA can be much more stable than the infectious capacity of the virus so that the presence of traces of RNA does not necessarily mean the risk of infection. Viral infectivity, on the other hand, is strongly affected by combined changes in temperature and relative humidity (see Sect. 3.1).

Finally, super spreading, i.e. single-infector contacts through which one person infects a disproportionate number of susceptible individuals, has been pointed out as a major factor in the transmission of the virus (Aschwanden 2020). Several factors that ease super spreading are tightly related to the environmental/biological fluid dynamics processes examined in this review. For instance, poorly ventilated indoor areas seem especially conducive to the virus’s spread because the chance of transmitting the pathogen in a closed environment is reasonably greater than in open-air space if anything because of turbulent dispersion. Clusters of COVID-19 cases occur mostly in indoor spaces, such as nursing homes, churches, food-processing plants, schools, shopping areas, worker dormitories, prisons and ships (Leclerc et al 2020). As the group of susceptible individuals in contact with a potential infector (i.e. improperly distanced (Sect. 4.5) and/or unprotected by personal protection equipment (Sect. 5.1)) grows in size, so does the risk of infecting a wider cluster. A role is attributable to the contact time as well, although exactly how much time a susceptible needs to pick up the contagion remains a yet unanswered question. The Center for Disease Control (CDC) of the US indicates 15 min of exposure as a guideline. This is a critical factor, in particular for essential workers who interact with large groups in situations primed for super spreading (Aschwanden 2020).

Needless to say, how people behave matters because certain activities make it easier to inhale respiratory gunk. In this review, we have reported about experimental studies where droplets are recorded flying when someone coughs or sneezes. Overall, the physics involved suggests that the isolated-drop emission picture is inadequate because respiratory liquid drops are formed and emitted embedded in a gas cloud whose presence is key to our understanding of range and persistence of pathogen-laden droplets. The images shown in Figs. 12, 13, 14 provide compelling evidence of this process. As we have seen in this Section, when you speak, you emit a tremendous amount of particles—speech emits more particles than normal breathing, and emissions also increase as people speak louder (Asadi et al 2019). Singing emits even more particles, which may partially explain superspreader events recorded after choir practice, or after fitness dance classes held in small rooms and all activities connected to exercising (Aschwanden 2020).

## Fluid dynamics of protection from airborne infection transmission

The recent developments of epidemiological models to analyze the spread of pandemic from COVID-19 in Italy (Gatto et al. 2020) has confirmed the crucial role played by drastic lockdown measures, to reduce the risks of infection transmission. However, the recent lifting of lockdown aimed at allowing economic and social recovery, along with the heavy burden of patients and medical staff died from COVID-19, does call for an assessment of the actual effectiveness of the measures that people are urged to adopt to reduce the risk of infection transmission. Two of them, namely wearing masks and insuring social distancing, have been widely implemented worldwide. We wish to contribute to the above assessment analyzing some consequences of the fluid dynamics of contagion outlined in the previous sections.

### Measures for facial protection

Masks are a commonly employed tool for facial protection. We are not interested, nor expert, on the details of the different types of masks available in the market. It suffices here to point out a few major distinct features of two most common typologies.

**Fig. 19:**
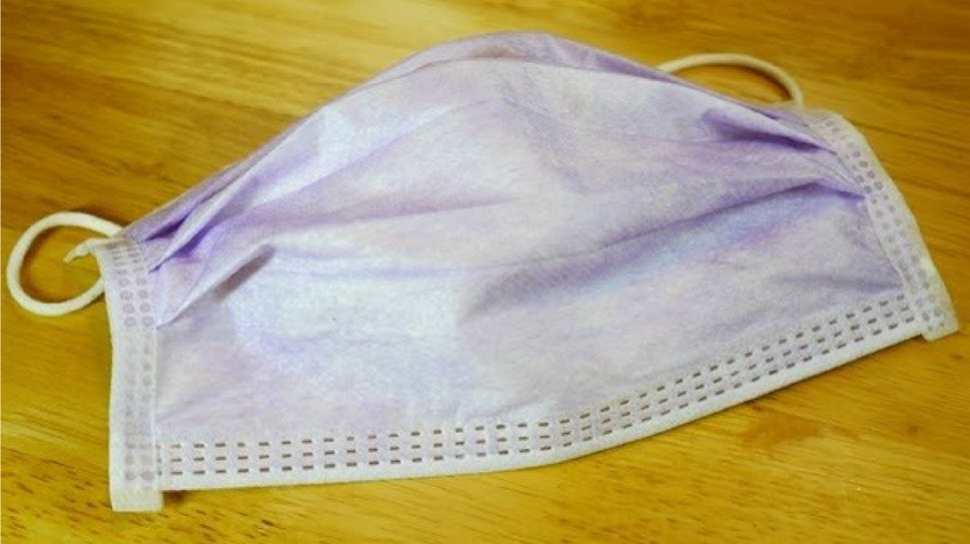
Surgical mask

Surgical masks (Fig. 19) are disposable tools that fit the face imperfectly. Individuals are expected to wear a surgical mask to prevent that infectious particles they release when coughing sneezing or simply speaking and breathing might affect other individuals in their neighborhood. The first masks systematically employed at the beginning of the twentieth century, employed cotton gauze and were worn by surgeons to prevent infections of patients during surgical operations. Progressively the use of surgical masks has been extended and their manufacture has evolved.

**Fig. 20:**
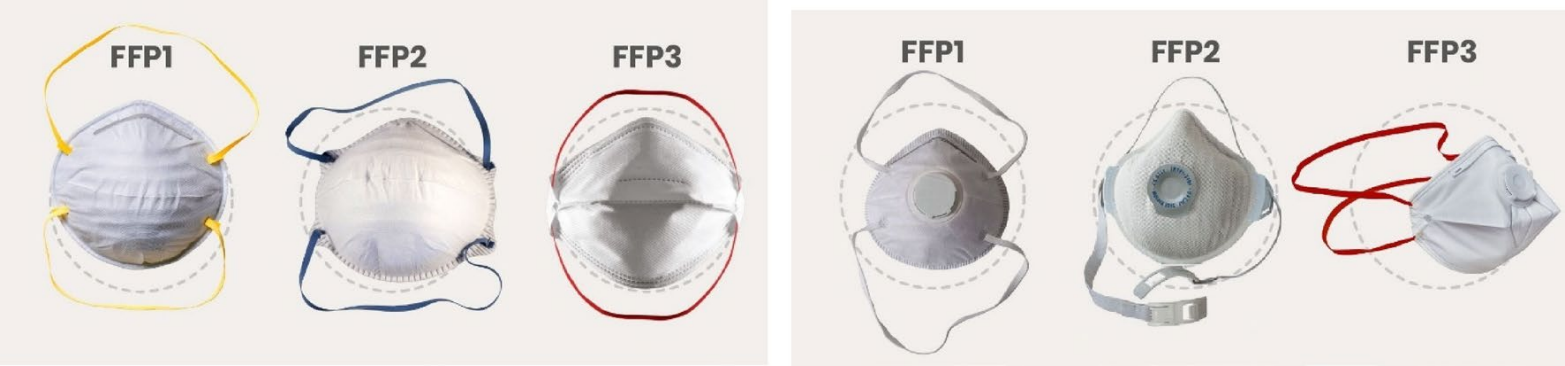
High protection masks devoid of (left) or equipped with (right) expiration valves

High protection masks (FFP*,* filtering facepiece particles) are tools designed to fit more tightly the human face. Their function is to filter the inhaled air flux, such to protect the person wearing the mask from contamination due to airborne infective particles. A systematic use of such masks began at the beginning of the last century. They were used by miners exposed to contamination from gases and dusts, by soldiers threatened by chemical weapons and by firefighters exposed to smoke and carbon monoxide. They have become a common protective tool for health workers in hospitals. In Europe they encompass three classes of protection (FFP1, FFP2 and FPP3) associated with three levels of filtering efficiency: at least 80%, 94% and 99% of airborne particles up to 0.6 μm size, respectively (Fig. 20). Note, that in the USA the equivalent of FFP2 masks is labeled N95. FFP masks may also be equipped with valves (Fig. 20). They make the use of masks more comfortable, as the valve allows one to expel the hot air and prevent its condensation. However, note that the valve radically changes the function of the mask, which no longer protects other people from infection transmitted by droplets exhaled by the individual wearing the mask!

Two features of the above description should be noted:

- first, high protection masks (devoid of valves) are believed to protect both the individual who wears them and people located nearby;
- second, the parameter adopted to measure the effectiveness of these devices is their filtering capacity. Masks should be able to capture infective particles in a wide size interval, from less than a micron up to more than 100 µm, with exhaled fluxes falling in the interval 10— 100 l/min. Research has long focused on the identification of the most appropriate material to be used to achieve these goals. In other words, the choice of a fibrous material is based on its filtering capacity (Konda et al. 2020). The latter depends on the diameter and thickness of fibres, as well as on their porosity and, possibly, their electric charge. These parameters control the various physical mechanisms, namely diffusion, interception, impact and electrostatic attraction that determine the particle-fiber contact, whereby particles are removed from the air flux. Typically, the dependence of the filtering efficiency on particle size exhibits a minimum for some particle size that define the ‘most dangerous’ particles.

EU directives provide rules for testing the efficiency of different masks, identifying ‘mask efficiency with ‘filtering efficiency’ (Prather et al. 2020). No reference is made to the need to account for issues of fit and leakage, although it has been ascertained that gaps (as caused by an improper fit of the mask) can result in a sharp decrease in the filtration efficiency, over 60% according to Konda et al. (2020). This important aspect is discussed in detail below.

#### Comparative evaluations of the efficiency of surgical versus FFP2 masks based on clinical data

We have reviewed a number of such evaluations available in the literature. However, we could not find conclusive evidence to support a generally agreed view: results appear to be strongly dependent on the type of virus and lead to a variety of different conclusions. Excerpts of such conclusions are reported below to support the latter statement.

Lee et al. (2008)

*Most of the tested N95 respirators and surgical masks in this study were observed to perform at their worst against particles approximately between 0.04 and 0.2 μm, which includes the sizes of coronavirus and influenza virus. The tested N95 respirators provided about 8–12 times better protection than the surgical masks.*

Johnson et al. (2009)

*On the basis of these preliminary findings, both surgical and N95 masks appear equally effective in preventing influenza dissemination from patients with confirmed influenza.*

Smith et al. (2016)

*…..our meta-analysis showed that there were insufficient data to determine definitively whether N95 respirators are superior to surgical masks in protecting health care workers against transmissible acute respiratory infections in clinical settings.*

Radonovich et al. (2019)

*Among outpatient health care personnel, N95 respirators vs medical masks as worn by participants in this trial resulted in no significant difference in the incidence of laboratory-confirmed influenza*

Long et al. (2020)

*The use of N95 respirators compared with surgical masks is not associated with a lower risk of laboratory-confirmed influenza. It suggests that N95 respirators should not be recommended for general public and non high-risk medical staff those are not in close contact with influenza patients or suspected patients.*

Bae et al. (2020)

*In conclusion, both surgical and cotton masks seem to be ineffective in preventing the dissemination of SARS-CoV-2 from the coughs of patients with COVID-19 to the environment and external mask surface.*

Leung et al. (2020)

*Surgical face masks significantly reduced detection of influenza virus RNA in respiratory droplets and coronavirus RNA in aerosols, with a trend toward reduced detection of coronavirus RNA in respiratory droplets. Our results indicate that surgical face masks could prevent the transmission of human coronaviruses and influenza viruses from symptomatic individuals.*

**Fig. 21:**
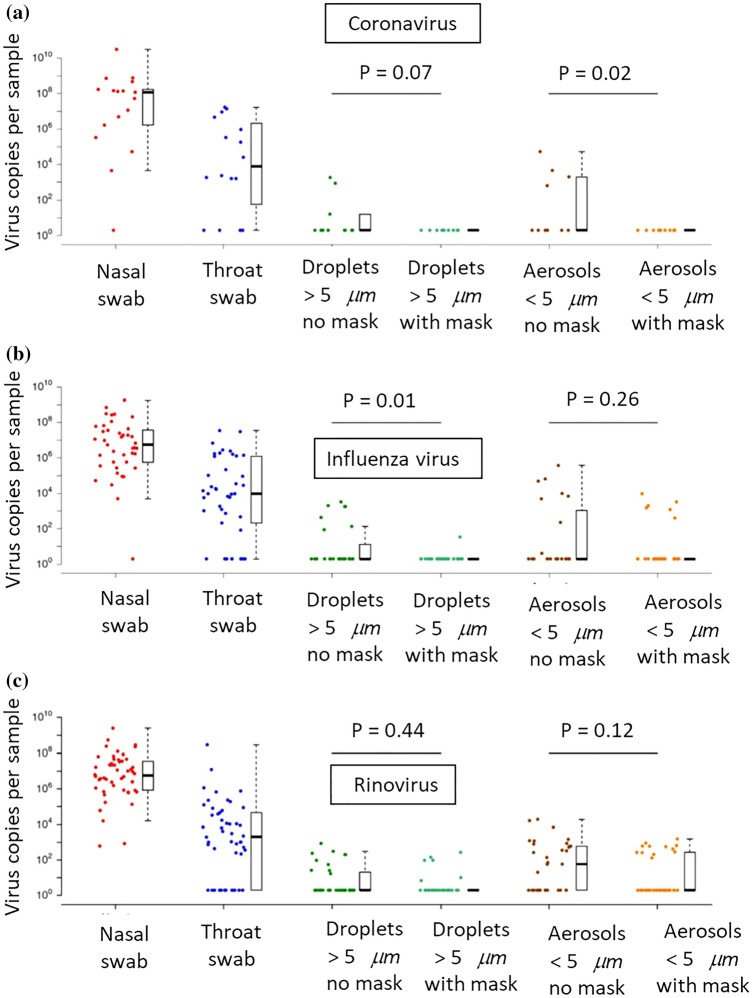
Efficiency of surgical masks in reducing the number of respiratory viruses exhaled in droplets of different sizes by symptomatic patients suffering from coronavirus (**a**), influenza (**b**) or rhinovirus (**c**). The figure plots the number of virus copies for each sample. Samples were collected from nasal swabs (red), pharyngeal swabs (blue), exhaled droplets (*d* > 5 μm) collected for 30 min from patients that did not wear (dark green) or wore (light green) a surgical mask and smaller droplets (*d* < 5 μm) collected for 30 min (brown no mask, orange with mask) (adapted from Leung et al. (2020)

The last study deserves special attention for the large number of patients involved in the experiments. From the initial group of patients examined (3.363), 246 were selected and their breathing exhalations were analyzed. 122 (124) of them did not (did) wear a surgical mask. Tests were made to ascertain the number of virus copies for each sample in nasal swabs, pharyngeal swabs, larger and smaller droplets exhaled when breathing. Results for patients wearing a surgical mask were compared with those for patients who did not wear a mask. Comparison is reported in graphical form in Fig. 21.

Results clearly suggest that the protective effect of wearing a surgical mask is strongly dependent on the size of the infected particle and the type of virus. The effect is strong for coronavirus (droplets of any size) and influenza (droplets of size higher than 5 µm). It is weak for rhinovirus (droplets of any size) and influenza (droplets of size lower than 5 µm).

#### Evaluation of the protective efficiency of respiratory masks through visualizations of exhaled flux.

Recently, Tang et al. (2009) employed sophisticated tools for the visualization of the exhaled cloud with its droplet load to analyze the exhalations of individuals who wore respiratory masks. This was an important step forward as it allowed one to investigate the real efficiency of various types of personal protective equipment, accounting for an effect usually overlooked, namely the role played by exhalation leakage due to an improper fit of the mask. Experiments employed Schlieren image technique, along with a high-speed video recording of the sequence of images and a PIV anemometer. Results are of great interest.

**Fig. 22:**
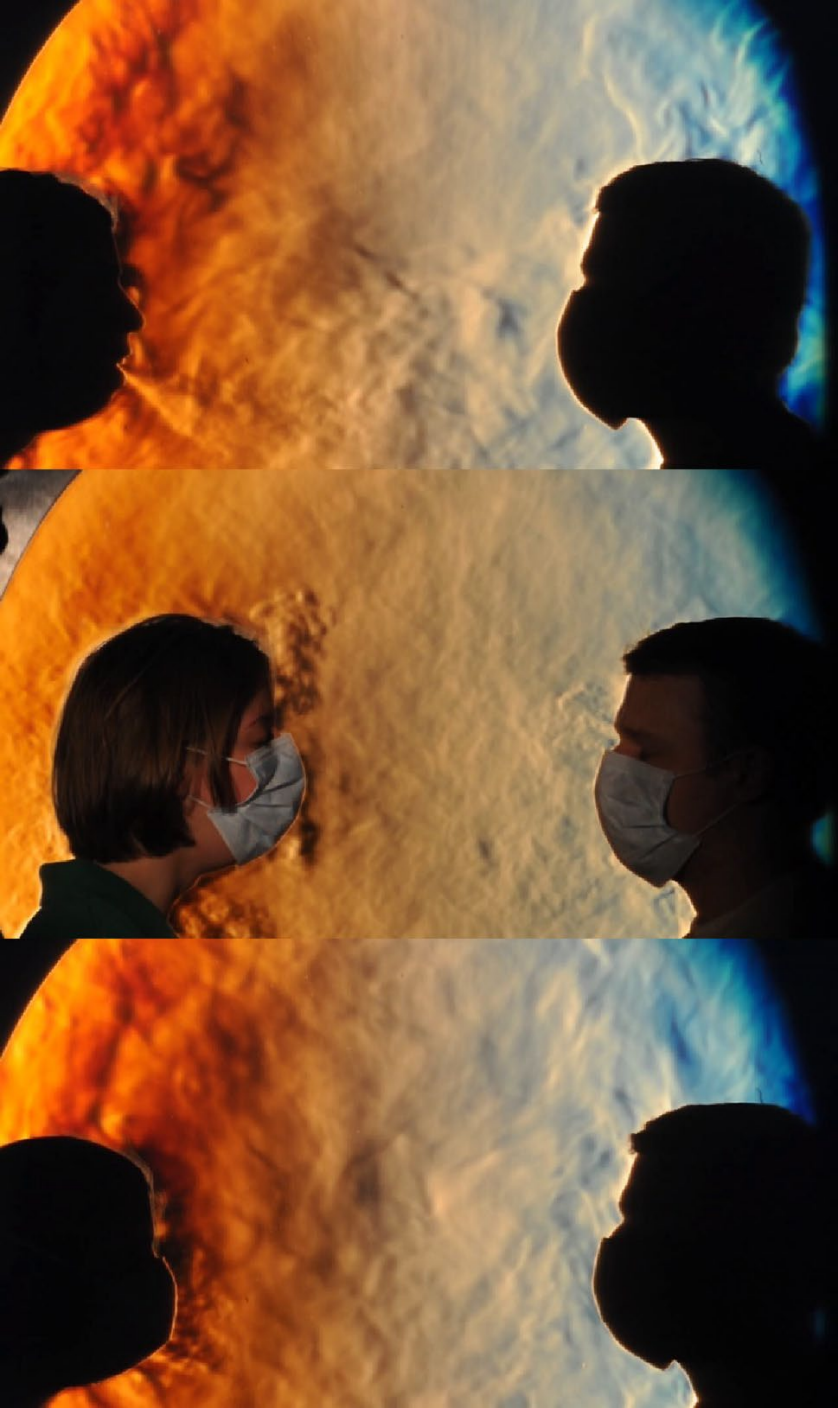
Schlieren images of two volunteers. The roughness of the Schlieren image is related to the turbulence of the emission, and visualizes the fluid flow of the emitted aerosols. In one instance, a person coughs without any mask protection (top), wearing a surgical mask (middle) and finally a FFP2 mask (bottom). The flow direction is inclined 308° downward in the top image. It has both vertical components (downward and upward) and lateral components that bypass the surgical mask in the middle image. The best fit of a FFP2 mask reduces the bypass flow but it increases the flux released through the mask. However, its weak speed limits the region affected by exhalations to the immediate neighborhood of the volunteer (images provided by Gary S. Settles)

Figure 22 provides a lateral view of two volunteers. One of them coughs. In the top image, the absence of any facial protection implies that the exhaled cloud, weakly inclined downward with respect to the horizontal, extends its influence through the whole region spanned by the image towards the second volunteer. Protection achieved wearing a surgical mask (middle image) educes the anterior flux and redirects it towards the edges of the mask, where it leaks through the mask-face gap. The better fit provided by a FFP2 mask lets the exhaled volume increase its pressure. As a result, the anterior flux through the mask also increases whilst the flux able to bypass the mask laterally is reduced. Moreover, as the speed of the exhaled flow is low, the latter is trapped into the weak human thermal plume that is known (e.g. Li et al. 2018) to be generated by the higher temperature of a human subject relative to the ambient air.

The study of Tang et al. (2009) has been recently extended by Viola et al. (2020), confirming the above observations and quantifying speed and direction of fluxes. Moreover, these authors have also considered the case of normal or intense breathing, the latter aimed at mimicking the effects of physical exercise. Seven different protective devices were analyzed, including a surgical and a FFP2 mask. Results may be summarized as follows:

- The cloud exhaled in the absence of any protective device has features similar to those reported by Tang et al. (2009) and Borouiba et al. (2014).
- The exhalation flux determined by cough is damped by a factor larger than 63% if FFP1 or FFP2 masks are worn. Moreover, the cloud reaches distances lower than 1/2 m or 1/4 m, respectively. If the mask does not fit the face perfectly, bypass fluxes are generated. However, they deviate upward moving little in the horizontal direction. On the contrary, wearing surgical masks or homemade masks, bypass fluxes are generated which disperse infected droplets in a region spanning various meters in a neighborhood of the source. Dispersion occurs in various directions, including the direction opposite to the main flux. This occurs both with intense breathing and with coughing.

The main conclusion of this study is that the efficiency of respiratory masks should not be evaluated only measuring their particle filtering capacity, but accounting also for the generation of secondary flows leaking through the gaps left at the edges of the mask due to its imperfect fit. This notwithstanding, the ability of masks to intercept most of the viral load should not be underestimated as surgical experience has demonstrated. The latter statement appears to be substantiated by a most recent assessment, funded by WHO, of data and metadata (Chu et al. 2020). This study aimed at estimating the interpersonal distance needed to avoid the transmission of infections among people that either wore or did not wear masks or eye protection devices. The main conclusion of this work concerns the case of 2647 empirical observations which suggest that social distancing, along with the use of masks and eye protection devices can provide a significant reduction of the risk of infection. This applies, in particular, to the case of N95 masks (the equivalent of FFP2 masks, cfr. Sect. 5.1), rather than to ordinary surgical masks. Eye protection is less effective to reduce the risk of infection from SARS-CoV-2, though it has been ascertained that it provides some marginal benefits.

**Fig. 23:**
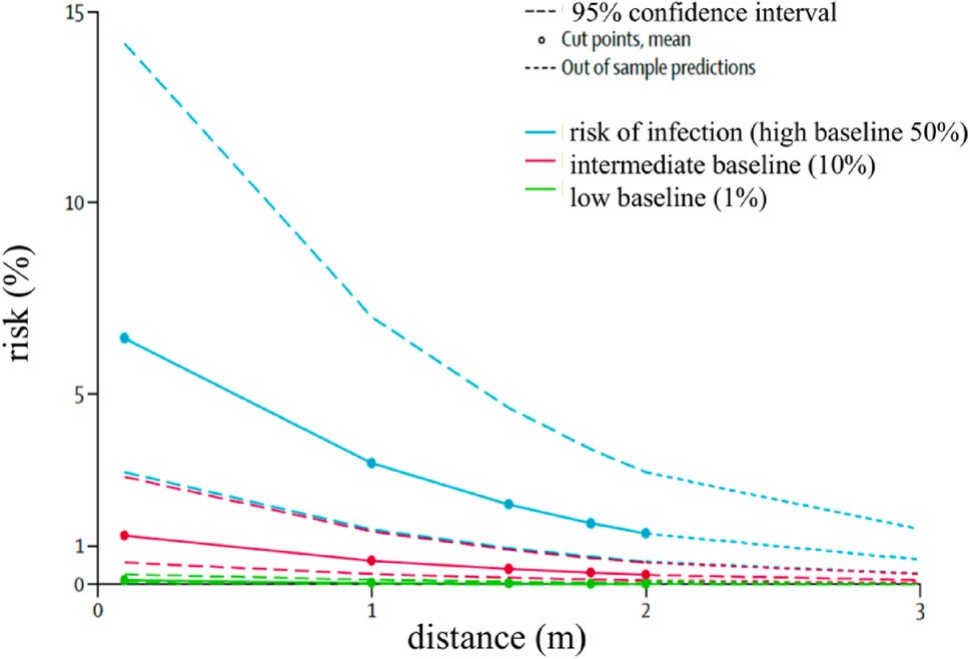
Variation of the absolute risk of infection from SARS-CoV-2 and SARS-CoV with distance infected-susceptible for given reference risks (baseline risk). The shift from a condition of high risk (high baseline risk) to an intermediate one, corresponding to the use of N95 masks or equivalent ones (adapted from Chu et al. 2020)

Chu et al. (2020) state that their results must be used with caution as they would need an appropriate series of randomized trials to formally check their actual validity. The main results of this study are summarized in Fig. 23, where the absolute risk of infection is plotted versus the distance infected-susceptible for various reference conditions (baseline risk) (note that the absolute risk is the larger between the pooling risk ratios and the adjusted odds ratios in Chu et al. 2020). The maximum distance in the plot is 3 m, though no actual data were available for this condition and the value was extrapolated from the randomized meta-analysis. As for the use of N95 masks or equivalent devices, their use by susceptibles exposed to infection decreases the risk of infection, corresponding to the shift from the high baseline to the intermediate baseline for infection. Finally, the comparative analyses of Chu et al. (2020) suggest that the efficiency of N95 is significantly higher than that of other types of masks, although this conclusion is in contrast with results of Bartoszko et al. (2020) based on four randomized trials.

### Fluid dynamics of social distancing

Let us close our discussion of the measures undertaken to protect the population from airborne infection transmission, with few notes concerning the well known issue of so-called ‘social distancing’. This issue stems from the guidelines released by WHO for the protection of health workers (World Health Organization 2020), where one reads:

“*Staff should be trained to protect themselves by maintaining a distance of at least 1 metre between themselves and travellers, at all times, (“social distancing”). Staff should also encourage travellers to maintain a more than 1 metre distance between themselves while waiting to cross the point of entry, including when completing entry forms.*”

The implicit assumption in the above statement is that a 1 m distance would ensure protection from infection associated with the dispersion of airborne infective droplets.

**Fig. 24:**
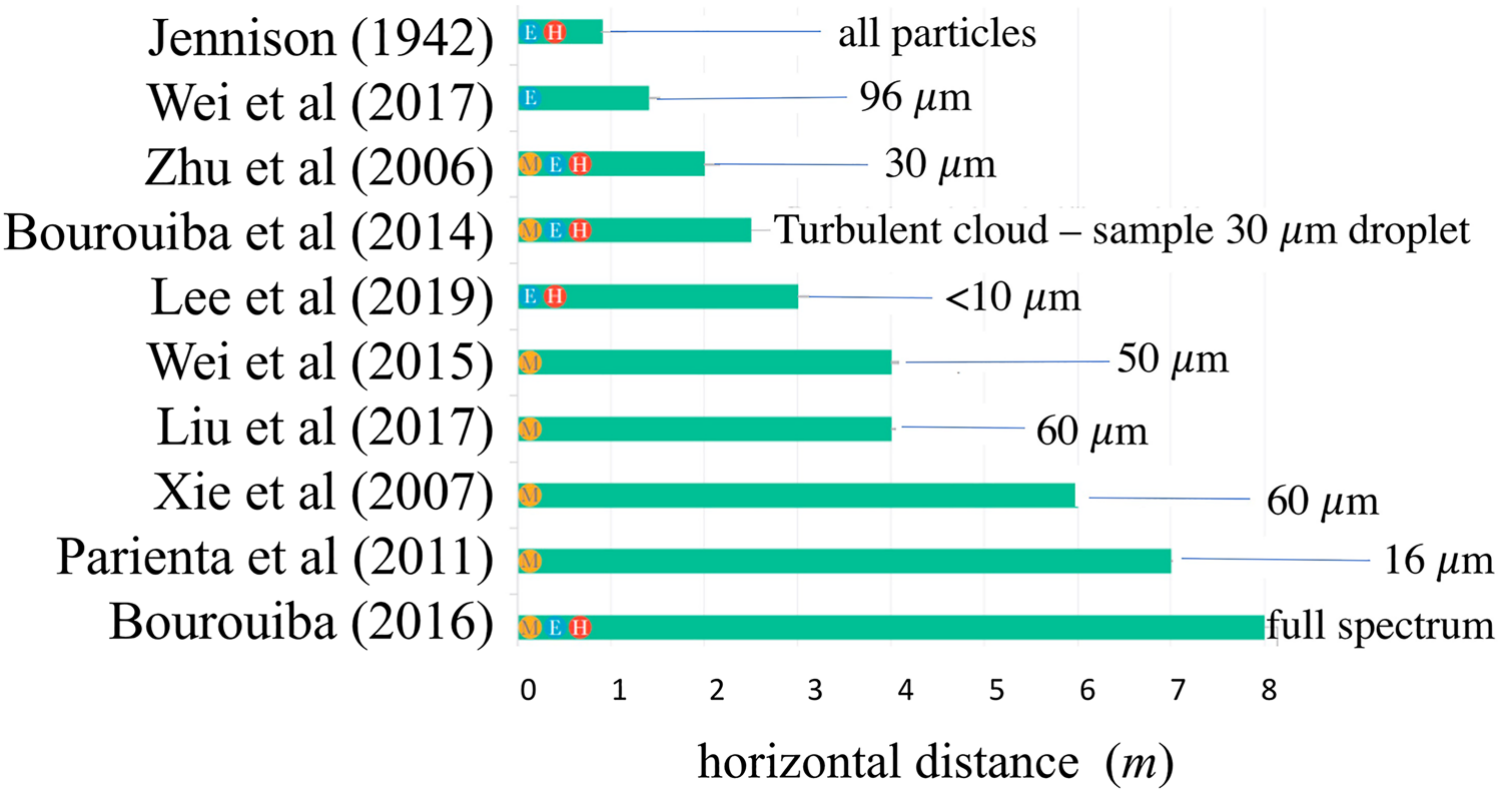
Prediction of the distance from the source reached by expiratory exhalations according to mathematical or numerical models, experimental observations or testing on patients. Note the large spread in the data, a possible consequence of the broad variability in the ambient conditions inside which the expiratory exhalations evolve (adapted from Bahl et al. 2020)

The state of the art that we have reviewed in this paper does not provide any scientific substantiation of this assumption. Similar conclusions emerge from a recent study (Bahl et al. 2020), where the current knowledge has been assessed. As illustrated in Fig. 24, numerical models and experimental observations of the most significant contributions provide a wide spectrum of predictions for the distance affected by respiratory exhalations, which invariably exceeds one meter.

The conclusion of the study reads:

“*We note that although the studies used very different methodologies and should be interpreted cautiously, they still confirm that the spatial separation limit of 1 meter (≈3 feet) prescribed for droplet precautions, and associated recommendations for staff at ports of entry [WHO, 2020], are not based on current scientific evidence*”**.**

This is a reasonable statement both when it recommends caution in adopting conclusions of studies whose theoretical foundations are sometimes questionable, as noted in the present review, and when it underlines the lack of scientific basis of WHO guidelines.

This notwithstanding, it is obvious that the probability of contagion decreases with distance. A quantitative estimate of this effect is given in Fig. 23. It shows that, in the absence of any protection, the risk of infection halves at one-meter distance and is still significant at 2 m distance. A strong risk reduction is obtained at a distance of several meters. Alternatively, adopting a suitable facial protection, the risk decreases strongly at much smaller distances. In other words, social distancing according to WHO guidelines, remains necessary, though not sufficient, measure to reduce the pandemic spread.

## Implications for the development of epidemiological models

In the context of the so-called non-pharmacological measures to contain the contagion, a special role belongs to large-scale measures of social distancing, including personal protection equipment (World Health Organization 2020); temporary closure of schools and universities; lockdown extended to public events or mass gatherings. These measures are obviously related to the general theme of this Review, i.e. the biological fluid dynamics relevant to the spread of infections. Several open issues in that field concern the connection between the fundamental transport mechanisms of viruses, and their survival in the environment. Others pertain the shedding of significant viral loads. One wonders what are the connections of the fundamental transport mechanisms that are necessary to the possible contagion and the macroscopic schemes that are needed to describe the strength and the diffusion of the infection at the community level, that is, epidemiological models of any kind (Anderson and May 2008).

The COVID-19 spread is often described, with different variants, by hierarchical compartmental models (systems of coupled ordinary differential equations), whose parameter estimation is carried out in a Bayesian framework. The dynamics of symptomatic, pre-symptomatic, or asymptomatic) after a latency time generated by the exposure to an infection source. The infection rate *λ* (at times termed force of the infection) is generally the product of the number of contacts between susceptible and infected individuals per unit of time, $c$ [1/*T*], and the probability of transmission of the disease per each contact, *β*. This parameter subsumes the effects of the biological fluid dynamics of the contagion dealt with here (e.g. Lipsitch et al. 2003; Tang et al. 2020). Current epidemiological models may be predictive about the expected number of contacts per unit time. However, the probability of transmission per contact, $\beta$, cannot be determined from first principles to date.

Large-scale processes like those involved in tracking and modeling human mobility and the containment of infections predictably affect the value of $c$. Values *λ* may be estimated by robust Bayesian methods via epidemiological modeling contrasting data, e.g. on case fatality counts unambiguously attributed to COVID-19 (Forsberg White and Pagano 2008; Flaxman et al. 2020; Gatto et al. 2020). Once data are contrasted by computations, and the latters prove capable of reproducing, say, spatial and temporal patterns within accuracy, the evolution of the force of the infection may be estimated reliably (Forsberg White et al. 2009). In this manner, it is possible to identify the role of the transmission mechanisms that were in place at the time of the estimation, like, e.g. in the latency period of a patient's infection. Similarly, substantial undocumented asymptomatic infections that facilitate the rapid spread of the SARS-CoV2 coronavirus may be inferred indirectly (Li et al. 2004; Li et al. 2020; Rothe et al. 2020). Backward schemes are also commonly used to determine the effective reproduction index, by retrospectively considering the distribution of delays between the manifestation of symptoms and death (Forsberg White and Pagano 2007; Wallinga and Lipsitch 2008). The empirical tracing of human mobility, i.e. the use of human mobility fluxes as data and not as a model to be tuned via the bulk of all other noise sources, is now possible on very large numbers via technologically advanced tools, for example, based on the tracking of mobile phones (e.g. Chinazzi et al. 2020; Ferretti et al. 2020; Pepe et al. 2020). This yields insightful establishments of one component of the force of the infection, the statistics of the number of contacts between susceptible and infected individuals per unit time,$c$.

An open problem of great interest and relevance is the theoretical prediction of the probability distributions of $\beta$ (the probability of transmission per contact), in particular as a function of the nature and severity of the symptoms of the infected donor. What is currently missing is an assessment of the conditions that determine: the distribution of near/far contacts in space and time (see Sect. 3.2); and the physical and biological characteristics of the particles emitted by the respiratory functions or simply speech (see Sect. 4.2).

**Fig. 25:**
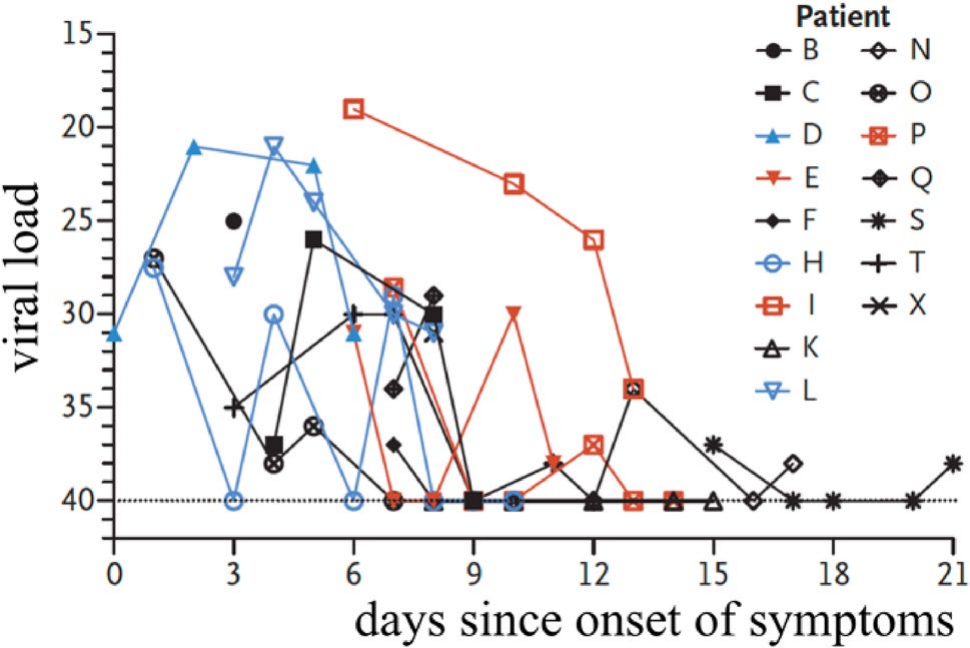
Viral load detected in nasal swabs obtained from patients infected with SARS-CoV. This is an example of direct measurement of the viral load cycle, here expressed in units of measure specific of a test system using RT-PCR (${C}_{\text{t}}$ value), immaterial to the evaluation of the cycle, days, that is of interest here (adapted from Zou et al. 2020)

Experimental and empirical evidence on the progression of COVID-19 with reference to the viral load of the infected varieties could usefully define an important condition for the emission models (and then the probability of contagion). A relevant empirical study has provided temporal profiles of the viral load in samples of oropharyngeal saliva from patients infected with SARS-CoV-2 (To et al. 2020). The result of the limited inference of other known acute respiratory syndromes proves remarkable. It suggests unmitigated prudence in the formulation of hypotheses usable for forecasts or scenarios. In fact, despite the limited evidence available to date, COVID-19 always seems to show the maximum viral load at the onset of the infection, thus justifying the rapid spread of the epidemic. A key parameter for determining the value of the distribution of $\beta$ could, therefore, be the age of the infection. i.e. days since the onset of symptoms in the infector that emit the saliva drops conveyed from the mouth to the surrounding environment within a puff of saturated air volume. The relative proportion of the various ages of infections might be calculated from compartmental models (Liu et al. 2020; Zou et al. 2020), thus somewhat configuring some kind of predictor–corrector method for determining the effective transmission rate. It is also useful to note that samples suited to the evaluation of viral loads are not invasive, and are usually acceptable to patients and healthcare personnel or groups at risk (e.g. RSA guests), natural attractors of systematic detection (Flaxman et al. 2020). Other studies in samples taken from the upper respiratory tract of infected patients in the nose and mouth (Zou et al. 2020) document the cycle of the viral load from onset to remission, providing a first outline of the boundary conditions for an emission model (see Sects. 3 and 4) (Fig. 25). The observations contained therein are relevant to an important epidemiological determinant: the viral load measured in asymptomatic patients is similar to that of patients with severe symptoms, suggesting the great potential contribution to the transmission of the epidemic by patients with no symptoms. This must be rooted in deeper explanations than available to date, possibly based on fluid dynamic transmission mechanisms, and biological mechanisms of pathogen survival in the environment.

Finally, the probability of transmission must describe any seasonality effect, at times assumed in analogy to that documented for other coronaviruses (Kissler et al. 2020). Seasonality may be relevant to interpret bio-fluid dynamics of the processes that occur at the drop–air interface in different climatic conditions (Sect. 3.3). It should be noted that the actual viral load transmitted with the infection, which has a significant impact on the course and case fatality counts of the disease (Cyranoski 2020), is determined by the boundary condition (the initial concentration of the emission. This is a function, today unknown, of the viral load of the type illustrated in Fig. 6) and of the dynamics of the diffusion process for large and small droplets (see Sect. 3). The relationship between the infectious load actually transmitted and the probability of contagion for each contact remains an open problem, potentially quite different from that of the toxicological thresholds that set a value below which the effect does not occur. If the SARS-CoV-2 infection threshold were not dose-dependent, as for many micro- and macro-parasitic diseases (Anderson and May 2008), the problem of dilution and opportunities for spreading the virus would have to be reviewed with the progress of empirical and experimental evidence. In analogy with problems of seasonality and of the actual duration of acquired immunity, which may or may not depend on the strength of the contracted infection.

## Discussion and conclusions

A number of open questions emerge from the present review. In our view, they might underpin our current inability to predict infection transmission and its prevention. They may be summarized by six questions, whose rationale and relevance are discussed below.

Q1: *Would a virus-carrying droplet undergoing evaporation (possibly shrinking down to its dry nucleus) maintain its infectivity?*

This question is related to the issue of stability of the virus within environments characterized by different turbulence levels and relative humidity. The relevant literature is reviewed in Sect. 3.1.

Incidentally, given the relevance of this problem and the continuous attention it has attracted for decades, it seems somewhat surprising that no conclusive assessment exists in the literature about the fundamental determinants of the persistence of virus infectivity in the environment. The hypothesis put forth in a WHO report (Sobsey and Meschke 2003) supports the view that, in general, viruses that are coated by a lipid membrane would retain their infectivity longer at low relative humidity. Uncoated viruses would instead be more stable in humid environments. However, this view is challenged by a number of counterexamples. This point has been made, in particular, by Yang and Marr (2012). They analyzed empirical evidence and the validity of previous hypotheses aimed at interpreting the correlation between virus stability and relative humidity in the environment. Among the relevant factors, one counts:

- removal of water molecules from the pericapsid, leading to virus inactivation;
- damage to viruses dividing on aerosol surfaces due to surface tension or shear stress effects;
- toxic effects of dissolved salts in droplets, possibly enhanced by the increase of their concentration owing to evaporation;
- conformation changes of surface glycoproteins present in coated viruses may be driven by pH variations of aerosols undergoing evaporation and compromise their infectivity.

A reference framework seems far from acknowledged. Sorting out the individual role and the collective effects of the various mechanisms remains an interdisciplinary research challenge. As documented in our Review, the latter open question naturally leads to following further open questions.

Q2: *is it possible to associate a probability distribution to the size of droplets dispersed in the two-phase flow exhaled by the various types of respiratory events?*

The large uncertainty associated with such an association (see Sect. 4.1) is certainly due in part to the different degree of sophistication in the instrumentation used by the different studies mentioned therein. However, this is only part of the explanation, and possibly not the most important one. For example, the visualizations by Scharfman et al. (2016) have shown that, at least in the case of the most violent respiratory events, the hypothesis that exhalations consist of the two-phase dispersion of droplets in a humid air stream is not entirely correct. Droplets form and evolve from complex liquid structures via fragmentation mechanisms whose modeling is still a major research challenge. Section 4.3 has highlighted that this issue is linked to a third open problem.

Q3: *How does the exhaled two-phase mixture form?*

The state of knowledge on this matter is still in its infancy. It is limited to the broad identification of the possible instability mechanisms of the mucus–air interface within the respiratory airways. Strong nonlinearities of the instabilities arising therein have not been modeled. They pose challenging problems of fluid dynamics, currently at the forefront of research on the fragmentation of liquid structures into droplets.

Q4: *How does the jet-puff cloud evolve in the near and far fields? How is this process affected by the presence/absence of secondary circulation induced by natural or forced ventilation?*

Recent progress in the advanced visualization of droplet clouds (Sect. 4.2) still await proper interpretation in the light of suitable turbulence models. These models must be capable of reproducing the time evolution of the velocity, temperature and relative humidity fields, jointly with settling velocity and evaporation of droplets (see Sect. 4.4). It is argued that only the availability of these tools, along with a solution to question Q1, will yield a comprehensive framework of the mechanics of contagion transmission and, therefore, of its possible prevention. In particular, we must assess whether forced ventilation may yield an effective danger to infection spreading, e.g. via possible contamination of aeration conduits, and if so to what degree. Note, in this respect, that significant traces of viral RNA have been detected in the inlets of aeration conduits in a hospital where SARS-CoV-2 patients were being treated (Santarpia et al. 2020).

Q5: *Can we offer reliable indications to decision makers about the efficacy of personal protection equipment and measures?*

The brief review outlined in Sect. 5 suggests that the WHO guidelines currently adopted for the measures of social distancing needs to be revisited. It emerges clearly that the currently suggested social distancing would require, to be truly effective, the joint use of facial protection tools able to significantly reduce the spreading distance of respiratory exhalations. Furthermore, facial protection devices must be maintained in place when speaking, as speaking is associated with an increase of exhalations. This issue has been reviewed in Sect. 4.2, where it has been emphasized that a verbal exchange is often an occasion of short-distance interaction among asymptomatic infected individuals and susceptible ones. In the absence of facial protection, current knowledge is unable to give binding indications on how to revise current protection criteria. An assessment will require that the open problems mentioned above be satisfactorily addressed.

Q6: *What experimental and theoretical validation may underpin a predictive definition of the probability of infection per single contact?*

The synthesis provided in this context could only yield a list of open issues, moving from measurements of the effective distancing per contact between an infected individual and a susceptible one. Evidence gathered on the viral load in nasal and throat cavities potentially determining the infective character of exhaled droplets will have to be used to provide boundary and initial conditions for the biological fluid dynamics model of pathogen release and transport processes. The current state of knowledge may provide reasonable schemes for the probability of contact under diverse conditions, but not on the probability of infection per contact. Together, these factors determine the force of the infection, which is currently estimated (say, in a Bayesian framework) from noisy data rather than from solid predictions based on fundamental principles.
